# LncR12304.1-miR1507c-*CsNADH-GOGAT* ceRNA module regulates amino acid biosynthesis in tea plant (*Camellia sinensis*)

**DOI:** 10.1093/hr/uhag014

**Published:** 2026-01-13

**Authors:** Zhiwei Liu, Yan Ma, Teng Wang, Qiuyue Chen, Dihan Yang, Yiqing Guan, Tianyuan Yang, Ming Zhao

**Affiliations:** College of Tea Science, Yunnan Agricultural University, Kunming, Yunnan 650201, China; College of Tea Science, Yunnan Agricultural University, Kunming, Yunnan 650201, China; College of Agronomy and Biotechnology, Yunnan Agricultural University, Kunming, Yunnan 650201, China; College of Food Science and Technology, Yunnan Agricultural University, Kunming, Yunnan 650201, China; College of Tea Science, Yunnan Agricultural University, Kunming, Yunnan 650201, China; College of Tea Science, Yunnan Agricultural University, Kunming, Yunnan 650201, China; State Key Laboratory for Tea Plant Germplasm Innovation and Resource Utilization, Anhui Agricultural University, Hefei, Anhui 230036, China; College of Agronomy and Biotechnology, Yunnan Agricultural University, Kunming, Yunnan 650201, China

## Abstract

Glutamate synthase (GOGAT) is crucial for nitrogen metabolism and amino acid biosynthesis in tea plants, yet the post-transcriptional regulation of *GOGAT* remains unclear. This study identified miR1507c as a direct interactor of *CsNADH-GOGAT*, confirmed by DLR assays and 5′RLM-RACE. Notably in tobacco, the relative luciferase activity in plants overexpressing *CsNADH-GOGAT* and co-expressing miR1507c + *CsNADH-GOGAT*m3 (mutant) were significantly higher than in those co-expressing miR1507c + *CsNADH-GOGAT*. Overexpression of miR1507c also significantly suppressed the expression of *CsNADH-GOGAT* and endogenous *NtNADH-GOGAT* homologs. Leveraging lncRNA sequencing, we screened lncR12304.1 as a ceRNA that regulates *CsNADH-GOGAT* by competitively binding to miR1507c. Cytoplasmic co-localization (validated by FISH) and direct interaction (confirmed by DLR assays) between lncR12304.1 and miR1507c were established. RNA pull down-qPCR further demonstrated miR1507c binding to both lncR12304.1 and *CsNADH-GOGAT*. The regulatory axis lncR12304.1–miR1507c–*CsNADH-GOGAT* was substantiated *in vivo*: (i) in tea roots/shoots under varying nitrogen treatments and following miR1507c suppression using Antagomir, and (ii) in tobacco via transient co-overexpression. Collectively, our results demonstrate the establishment of this ceRNA network and its role in regulating glutamate and theanine biosynthesis.

## Introduction

ceRNA (competitive endogenous RNA) mainly includes circular RNA (circRNA), long non-coding RNA (lncRNA), pseudogenes, etc. It can competitively bind to miRNA through miRNA response elements (MREs) to regulate the mRNA expression of target genes. The above miRNA-centered post-transcriptional control mode of gene expression is called the ceRNA regulatory network [1], which has been confirmed to play an important role in human, animal, and plant life activities and metabolism [2, 3]. Compared to humans and animals, research on the ceRNA network in plants, especially woody plants, is relatively lagging. Except in model plants, the ceRNA networks in other plants are mostly in the preliminary prediction and identification stage, lacking in-depth functional validation. Now, the lncRNA and circRNA functions of regulating growth and development, stress resistance have been increasingly confirmed in more plants [4, 5]. For example, those non-coding RNAs involved in regulating the effectiveness of plant nitrogen utilization [6, 7], lncRNA-mediated ceRNA network responds to nitrogen stress [8] and different forms of nitrogen nutrition [9]. Therefore, the ceRNA network has been confirmed to be involved in regulating plant nitrogen metabolism, but related research is still in its infancy.

In higher plants, the GS/GOGAT cycle, composed of glutamate synthase (GOGAT) and glutamine synthase (GS), is the main pathway for NH_4_^+^ assimilation, and GOGAT is the rate-limiting enzyme in this pathway. GOGAT is divided into ferritin (Fd) and NADH reduction type, which are mainly responsible for ammonia assimilation in plant leaves and roots, respectively [10, 11]. Research has shown that GOGAT is closely related to nitrogen utilization efficiency and carbon-nitrogen balance [12, 13]. Therefore, GOGAT is a key enzyme in plant nitrogen metabolism pathways, playing an important role in ammonia assimilation and amino acid biosynthesis. At present, *GOGAT* genes have been cloned from multiple species, and their expression showed tissue-specific, influenced by light, nitrogen, and various plant regulatory factors [14]. Due to long sequence of *GOGAT* (cDNA sequence generally exceeding 6 kb), its functional research is relatively scarce compared to *GS*. The overexpression/inhibition of *GOGAT* mainly focuses on *Arabidopsis*, tobacco, and rice, poplar [11, 15]. Research has found that transcription factors, such as NAC, DF1, and MYB30 can regulate the expression of *GOGAT* at the transcriptional level [14, 16]. However, at the post-transcriptional level, there are few reports on non-coding RNA and ceRNA networks that target the regulation of *GOGAT* expression.

In tea plant (*Camellia sinensis*), amino acids are important metabolites in the nitrogen cycle, accounting for approximately 1% to 4% of the dry weight of tea shoots. They are essential flavor and functional components of tea, especially the L-theanine, which is characteristic amino acid in tea plant and biosynthesized from glutamate and ethylamine [17]. Therefore, elucidating the molecular regulatory mechanism of key genes involved in amino acid metabolism is an essential foundation for breeding the tea varieties with abundant amino acids. In recent years, multiple transcription factors involved in regulating amino acid biosynthesis, such as MYB [18], WRKY40 [19], as well as amino acid transporters AAP [20], LHT [21], have gradually been identified. In contrast, there are few reports on hierarchical regulation of non-coding RNAs, such as lncRNA, circRNA, in the amino acid metabolic network [22]. Among them, miRNAs targeting amino acid synthesis-related structural genes *GS*, A*DC*, *GDH*, and *ALT* in tea plants, as well as lncRNA targeting *TS*, have been preliminarily predicted [7, 23, 24], but their mechanisms of action need to be validated. At present, it has been reported that the ceRNA network in tea plant is mainly related to response to cold resistance [25], whitening [26], pests, and diseases [2, 27]. However, the study of ceRNA networks involved in regulating the metabolism of major flavor substances such as amino acids is still in its infancy, and there have been no reports on the regulation of amino acid metabolism by tea ceRNA networks.

In this study, our objective is to systematically analyze the mechanism by which the ceRNA network “lncRNA-miRNA-*CsNADH-GOGAT*” regulates amino acid metabolism in tea plants. Based on our reported transcriptome and microRNA data [23], we screened the specific miRNA targeting *CsNADH-GOGAT* and identified their interaction by dual-luciferase reporter (DLR) assay, 5′RLM-RACE, and transient transgenic identification. Then the lncRNAs as ceRNA, regulating *CsNADH-GOGAT* by mediating miRNA, were preliminarily screened through the lncRNA sequencing. The ceRNA regulation network was further verified by *in vitro* validation via fluorescence in situ hybridization (FISH), DLR assay, RNA pull-down technologies, and *in vivo* validation in tea plant and tobacco. The significance of our study lies in: (i) revealing the molecular paradigm of ceRNA regulation of amino acid synthesis in tea plants and improving the “RNA layer control” theory of nitrogen metabolism; (ii) Promote the study of secondary metabolism ceRNA mechanisms in tea plants, providing a scientific theoretical basis for targeted breeding and improvement of high amino acid tea varieties.

## Results

### MiR1507c targets and regulates *CsNADH-GOGAT*

**Figure 1:**
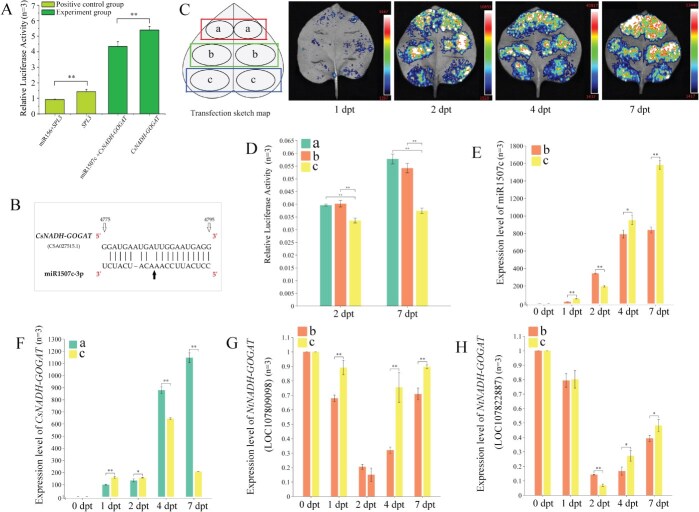
Identification of interaction between miR1507c and *CsNADH-GOGAT*. (A) Luc/Ren fluorescence ratio analysis of dual-luciferase reporter assay; (B) The splice site verification of miR1507c for *CsNADH-GOGAT* by 5′RLM-RACE; (C) The fluorescence photographic images of tobacco leaves overexpressing *CsNADH-GOGAT* (a), *CsNADH-GOGAT*m3 + miR1507c (b), and *CsNADH-GOGAT* + miR1507c (c) at 1, 2, 4, 7 days post-transfection (dpt); (D) Luc/Ren fluorescence ratio analysis of a, b, c at 2 and 7 dpt; (E) The miR1507c expression levels of b and c; (F) The *CsNADH-GOGAT* expression levels of a and c; (G, H) The *NtNADH-GOGAT* homologs expression levels of b and c. *n* = 3: three biological replicates.

Based on integrated analysis of reported transcriptome and microRNA data, *CsNADH-GOGAT* as a putative target gene of gma-miR1507c-3p (Abbrev. miR1507c) was identified in tea plants exposed to varying nitrogen levels. Dual-luciferase reporter (DLR) assays revealed a significantly reduced Luc/Ren ratio in tobacco co-expressing *CsNADH-GOGAT* and miR1507c compared to those expressing *CsNADH-GOGAT* alone (Fig. 1A), demonstrating their putative targeting relationship. Furthermore, 5′RLM-RACE validated cleavage sites within ORFs region of *CsNADH-GOGAT* at the 11th to 12th base site of miR1507c (Fig. 1B).

We next monitored relative luciferase activity and gene expression profiles at 1, 2, 4, and 7 days post-transfection (dpt) in tobacco leaves. *CsNADH-GOGAT* overexpression produced the highest relative luciferase activity, while co-expression of miR1507c + *CsNADH-GOGAT* yielded the lowest. Notably, fluorescence signals intensified progressively over time across all three experimental groups (Fig. 1C; Fig. S1). At both 2 dpt and 7 dpt, the LUC/REN ratio in leaves transformed with *CsNADH-GOGAT* alone or with miR1507c + *CsNADH-GOGAT*m3 showed no significant difference; however, both were significantly higher than in leaves co-expressing miR1507c + *CsNADH-GOGAT* (Fig. 1D). Concurrently, miR1507c levels increased progressively in tobacco leaves co-expressing miR1507c with either *CsNADH-GOGAT* or *CsNADH-GOGAT*m3 (Fig. 1E). *CsNADH-GOGAT* transcript levels gradually increased when expressed alone, but were significantly lower than in miR1507c + *CsNADH-GOGAT* co-expression samples at 4 dpt and 7 dpt (Fig. 1F). Interestingly, expression of two endogenous *NtNADH-GOGAT* homologs was significantly suppressed by miR1507c, reaching minimal levels at 2 dpt (Fig. 1G and H).

### Sequencing and screening of lncRNA interacting with miR1507c

**Figure 2:**
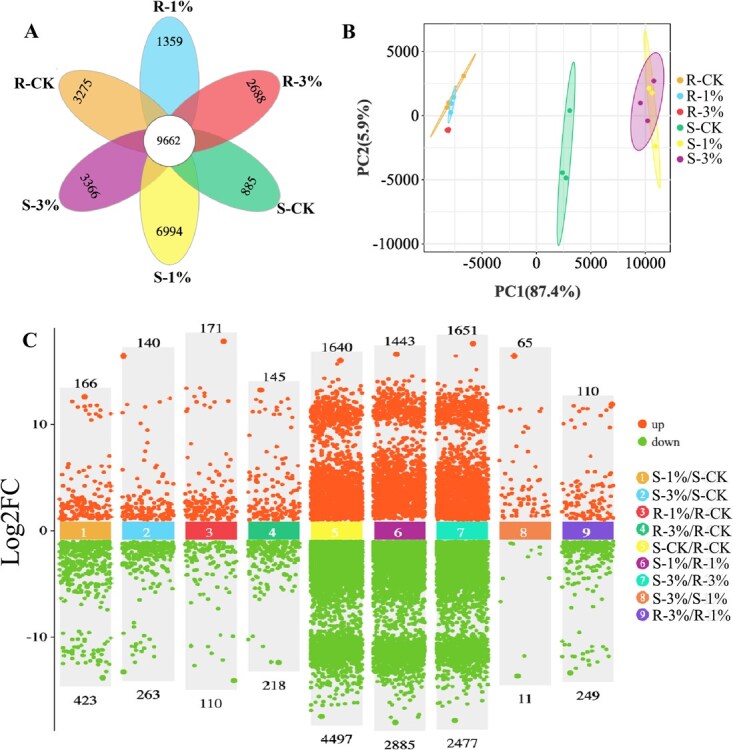
LncRNAs analysis under different nitrogen levels in tea shoot and root. (A) Venn analysis of LncRNA quantity; (B) PCA analysis of LncRNA (TPM ≥1) composition; (C) Difference analysis of LncRNA in different comparison group (FDR < 0.05, |log₂FC| > 2); S/R-CK: Tea shoots/roots treated with no urea as control, S/R-1%: Tea shoots/roots treated with 1% urea, S/R-3%: Tea shoots/roots treated with 3% urea.

A total of expressing 53 206 lncRNAs were identified across all samples in response to nitrogen treatments (Table S1). Under nitrogen (N) supply, lncRNA quantity significantly declined in tea roots but increased in shoots (Fig. 2A). Concomitantly, lncRNA compositional profiles showed greater difference in tea shoot than that in root under different N levels treatment (Fig. 2B). N treatment exerted stronger effects on tea shoots than roots for both lncRNA composition and abundance.

With the N supplementation increased, the differentially expressed lncRNAs (DELs) amount in tea roots increased but in shoots decreased significantly (Fig. 2C). Additionally, the DELs identified through inter-organ comparisons (shoots vs. roots) substantially outnumbered those detected in intra-organ comparisons under differential N levels. Targeted relation-based screening preliminarily identified 62 lncRNAs as potential targets of miR1507c (Table S2), from which four candidates with binding energy > −23.5 kcal/mol and minimum free energy (MFE) ratio > 70% were selected for further experimental validation.

### LncR12304.1 targets and interacts with miR1507c

**Figure 3:**
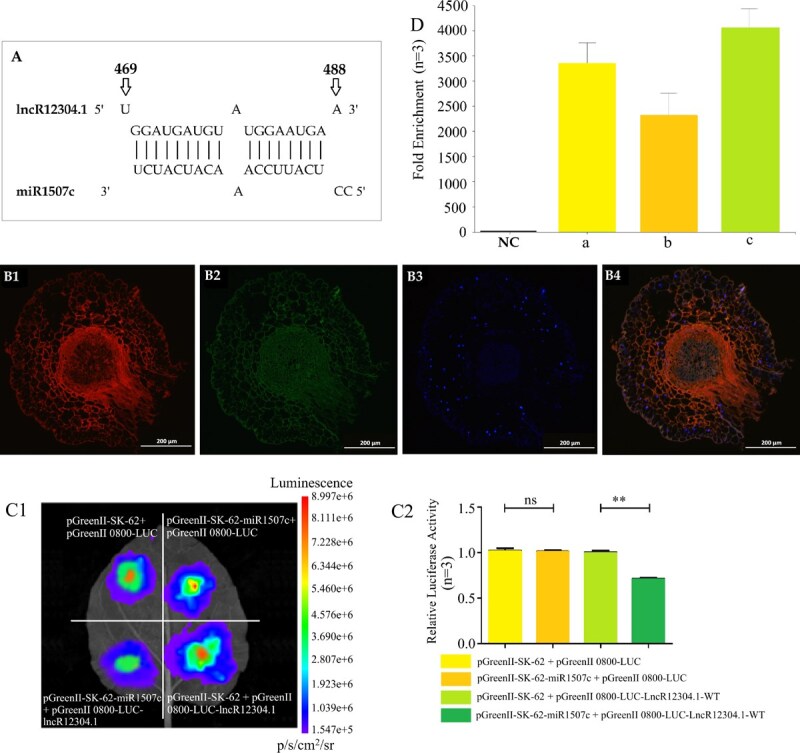
Identification of interaction between lncRNA and miR1507c. (A) The binding site prediction of miR1507c to lncR12304.1 (MFE = −29.2 kcal/mol); (B) Co-localization identification by FISH. (B1) Red fluorescence: miR1507c, (B2) Green fluorescence: lncR12304.1, (B3) Blue fluorescence: nucleus, (B4) Superposition of three fluorescein; (C) The adsorption confirmation of miR1507c to lncR12304.1 and *CsNADH-GOGAT* by RNA pull down - qPCR. NC: negative control, a/b: fold enrichment value of lncR12304.1 using the amplification primers designed by GGATGATGT/TGGAATGA sequence, c: *CsNADH-GOGAT* abundance; (D) Fluorescence photography (D1) and relative luciferase activity (D2) analyses of DLR assay. ^**^P < 0.01, *n* = 3: three biological replicates.

Given its high abundance and significant N responsiveness among the four candidates, lncR12304.1 was selected to investigate its interaction with miR1507c. TargetScan analysis predicted the miR1507c binding site at nucleotides 469–488 of lncR12304.1 (Fig. 3A). Using the transverse section of tea root, FISH confirmed obvious spatial co-localization of miR1507c (red fluorescence) and lncR12304.1 (green fluorescence) predominantly within cytoplasmic compartments, as evidenced by high signal overlap (Fig. 3B). These observations support potential possibility of combination between miR1507c and lncR12304.1. RNA pull down-qPCR analysis demonstrated that lncR12304.1 was significantly enriched by the miR1507c probe, showing fold-changes of 3.34 × 10^3^ and 2.32 × 10^3^ relative to negative controls, while *CsNADH-GOGAT* exhibited an enrichment of 4.07 × 10^3^-fold. These results confirm the specific physical binding between miR1507c and both lncR12304.1 and *CsNADH-GOGAT* (Fig. 3C).

To further validate the direct binding of lncR12304.1 to miR1507c, dual-luciferase reporter assays were performed in tobacco leaves co-transfected with the respective overexpression vectors. Compared with the three control groups, the fluorescence imaging revealed complete signal quenching when co-expressing lncR12304.1 and miR1507c (Fig. 3D1). Quantitatively, relative luciferase activity showed no significant difference between (i) pGreenIISK-62-miR1507c + pGreenII0800-LUC (scrambled control) and (ii) pGreenII62-SK + pGreenII0800-LUC (empty control). However, co-expression of miR1507c with lncR12304.1 caused significant reduction in luciferase activity compared to the scrambled control (pGreenII62-SK + pGreenII0800-LUC-lncR12304.1-WT) (Fig. 3D2). These results demonstrate that miR1507c directly binds lncR12304.1, leading to targeted suppression of firefly luciferase reporter expression.

### LncR12304.1 as ceRNA regulates *CsNADH-GOGAT* by mediating miR1507c *in vivo*

#### In tea plant under different N levels treatment

**Figure 4:**
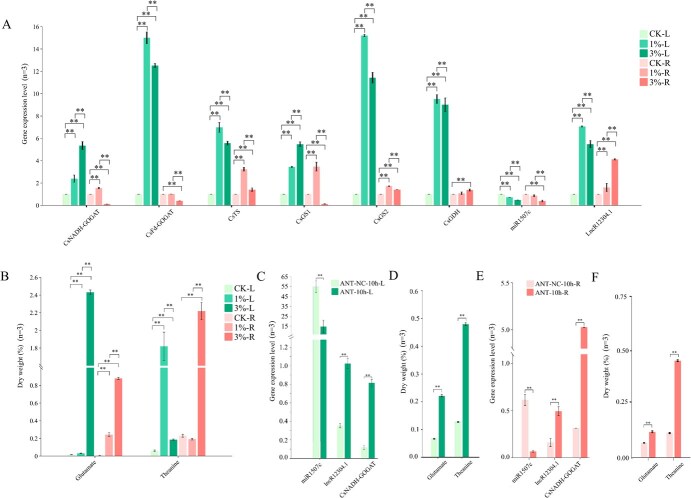
Expression profiles of miR1507c, *CsNADH-GOGAT* and LncR12304.1 (A, C, E), and their regulation effect on l-glutamate and l-theanine accumulation (B, D, F). (A, B) under different nitrogen levels treatment; (C–F) under Antagomir treated for 10 h. ^**^P < 0.01, *n* = 3: three biological replicates.

To validate the lncR12304.1 as a ceRNA that regulates *CsNADH-GOGAT* expression through competitively binding to miR1507c *in vivo*, transcript profiles of key structural genes in L-glutamate and L-theanine biosynthesis were analyzed in tea shoots and roots under graded N treatments, including *CsNADH-GOGAT*, *CsFd-GOGAT*, *CsTS*, *CsGS1*, *CsGS2*, and *CsGDH*. Nitrogen fertilization significantly upregulated all genes, except in roots where *CsNADH-GOGAT*, *CsFd-GOGAT*, and *CsGS1* were suppressed at 3% N treatment. Concurrently, miR1507c expression decreased dose-dependently with increasing soil N, while lncR12304.1 levels exhibited inverse trends (Fig. 4A). This demonstrates miR1507c’s negative regulation and lncR12304.1’s positive modulation of *CsNADH-GOGAT* across most conditions. Correspondingly, L-glutamate content increased progressively with N availability. L-theanine accumulation peaked in shoots at 1% N but required 3% N for maximal production in roots (Fig. 4B).

#### In tea plant with miR1507c transient suppression

Sampling tea shoots and roots, we performed transient suppression of miR1507c using Antagomir. Following 10 h post-treatment, transcript levels of lncR12304.1 and *CsNADH-GOGAT* were quantified in both tea shoots and roots. Consistently across tissues, miR1507c downregulation significantly upregulated both lncR12304.1 and *CsNADH-GOGAT*. Statistical analysis revealed negative correlation between miR1507c and lncR12304.1/*CsNADH-GOGAT* expression, while positive correlation between lncR12304.1 and *CsNADH-GOGAT* (Fig. 4C and E). Meanwhile, the glutamate and theanine contents were significantly increased (Fig. 4D and F). Concomitantly, L-glutamate and L-theanine concentrations increased significantly (*P* < 0.05), consistent with enhanced *CsNADH-GOGAT* abundance.

#### In transient transgenic tobacco

**Figure 5:**
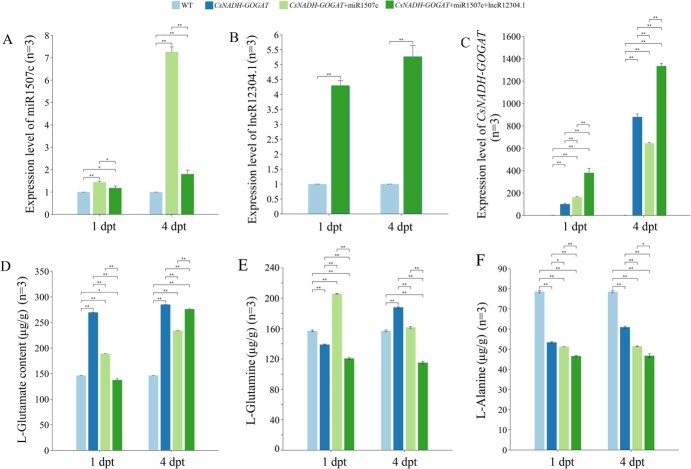
Expression profiles of miR1507c (A), *CsNADH-GOGAT* (B), LncR12304.1 (C) and abundance of l-glutamate (D), l-glutamine (E), l-alanine (F) in transient transgenic tobacco. Note: Light blue column: wild type tobacco; Dark blue column: tobacco overexpressing *CsNADH-GOGAT*; Light green column: tobacco co-overexpressing *CsNADH-GOGAT* and miR1507c; Dark green column: tobacco co-overexpressing *CsNADH-GOGAT*, miR1507c, and LncR12304.1; ^*^P < 0.05, ^**^P < 0.01, *n* = 3: three biological replicates.

In transgenic tobacco where lncR12304.1 was jointly overexpressed with miR1507c and *CsNADH-GOGAT*, the miR1507c abundance was significantly (*P* < 0.05 at the first dpt; *P* < 0.01 at the fourth dpt) lower compared to tobacco co-overexpressing miR1507c and *CsNADH-GOGAT* (Fig. 5A and B). Conversely, *CsNADH-GOGAT* expression was significantly higher in the presence of lncR12304.1 overexpression (Fig. 5C). Compared to the expression profile in transgenic tobaccos of the DLR assay, the levels of miR1507c and *CsNADH-GOGAT* increased similarly over time when they were overexpressed. Interestingly, the co-overexpression of miR1507c and *CsNADH-GOGAT* led to a significantly (*P* < 0.01) depressed *CsNADH-GOGAT* expression level specifically at the fourth dpt, which was also consistent with the findings described in the DLR assay. In contrast, *CsNADH-GOGAT* expression significantly increased (*P* < 0.01) when lncR12304.1 overexpression was included.

Overexpression of *CsNADH-GOGAT* significantly enhanced L-glutamate accumulation, whose profiles mirrored *CsNADH-GOGAT* expression patterns particularly at the fourth dpt (Fig. 5D). Additionally, the L-glutamine accumulation was also influenced under transformation, and showed almost opposite profile corresponding to L-glutamate (Fig. 5E). The abundance of L-alanine, which generates ethylamine as another substrate for the L-theanine synthesis, significantly reduced (*P* < 0.01) in transgenic lines (Fig. 5F). These results demonstrated that lncR12304.1 probably functions as a ceRNA, regulating *CsNADH-GOGAT* expression by competitively absorbing miR1507c and influencing the amino acids accumulation correspondingly.

## Discussion

LncRNA, defined as a non-coding RNA exceeding 200 nucleotides (nt) in length, are ubiquitous across eukaryotic species and play indispensable roles in genetic regulation. LncRNAs modulate gene expression through diverse mechanisms, primarily categorized as epigenetic, transcriptional, and post-transcriptional regulation [28]. A well-characterized post-transcriptional mechanism involves lncRNAs functioning as ceRNAs, which bind to miRNAs to influence target gene expression [29]. In tea plants, lncRNAs responsive to various stresses, including salt resistance [30], nitrogen application [7], temperature stress [31], and tea leaf spot disease [32–35], have been preliminarily screened. Given the critical importance of characteristic secondary metabolites for tea quality, lncRNAs associated with aroma formation in black tea [36] and metabolites accumulation during the solar-withering process of oolong tea [37] have also garnered significant attention. Recently, a specific ceRNA network (LncRNA81246-miR164d-*NAC1*) regulating resistance against tea leaf spot was experimentally verified [27]. Nevertheless, lncRNA research in tea plants remains nascent, especially the lncRNAs as ceRNA regulating the key genes involved in flavor substance like amino acids require further validation through functional studies.

Our previous study demonstrated that the CsNADH-GOGAT, a key enzyme in the amino acid metabolic pathway, is crucial for the accumulation of glutamate and theanine in tea plants [10]. In this study, we constructed and experimentally validated a ceRNA regulatory module dominated by an lncRNA that targets *CsNADH-GOGAT*, revealing its responsiveness to varying nitrogen levels. Building on our prior prediction [23], we confirmed that gma-miR1507c-3p, a member of the miR1507 family first identified in *Glycine max* (L.) Merr [38], binds to *CsNADH-GOGAT* and also regulate the homologous *NADH-GOGAT* in tobacco [39]. Originally considered legume-specific, miR1507 regulates saline-alkali tolerance [40] and *Phytophthora sojae* resistance [41] in soybean, and drought response in *Medicago truncatula* [42], while our findings extend its function to amino acid regulation in tea plants. MiR1507, as a ceRNA, is competitively bound by lncRNA and *CsNADH-GOGAT* and participates in regulating nitrogen assimilation pathways. Furthermore, experimental evidence indicates that this regulatory module is biologically active and contributes significantly to amino acid metabolism, with functional conservation being observed in both roots and leaves.

LncRNA sequencing revealed numerous DELs under varying N application levels. Previous studies demonstrated that lncRNAs respond to changes in N concentration in the roots of tea plants [7] and Moso bamboo [8]. Our study extends this finding, showing that lncRNAs in both tea roots and shoots are influenced by N supply. As N supplementation increased, the amount of DELs exhibited opposite trends in roots versus shoots. Notably, the number of DELs within organs (intra-organ) far exceeded those between organs (inter-organ), clearly illustrating the tissue specificity of lncRNA expression [30]. This specificity is likely associated with differences in transcription factor motif density and SNPs within promoters [43]. Among the DELs significantly responsive to N and predicted to interact with miR1507c, we selected lncR12304.1 for further experimental validation. This selection was based on its predominant cytoplasmic localization, as cytoplasmic lncRNAs can directly bind miRNAs and function as molecular sponges [44].

Subsequently, DLR assays confirmed targeted binding between miR1507c and lncR12304.1. RNA pull-down coupled with qPCR further demonstrated that miR1507c exhibits binding affinity not only for lncR12304.1 but also for *CsNADH-GOGAT*. This observation aligns with the molecular sponge mechanism of miRNAs within ceRNA networks [44, 45], which was further substantiated *in vivo*. Upon examining tea shoots and roots under varying N levels, the abundance of miR1507c was predominantly negatively correlated with both lncR12304.1 and *CsNADH-GOGAT* levels. Conversely, lncR12304.1 and *CsNADH-GOGAT* exhibited a positive correlation. In tea shoots, increased levels of glutamate and theanine were attributed to the activity of the ceRNA module lncR12304.1-miR1507c-*CsNADH-GOGAT*, along with the heightened abundance of nitrogen metabolism genes (*CsFd-GOGAT*, *CsTS*, *CsGS1*, *CsGS2*, *CsGDH*). Notably, in tea roots exposed to 1% N, glutamate levels were elevated through the coordination of the ceRNA module with gene activity (*CsTS*, *CsGS1*, *CsGS2*). Despite the lack of established ceRNA regulation in tea roots under 3% N, the expression of *CsTS*, *CsGS2*, and *CsGDH* remained significantly higher than in the control, coinciding with elevated glutamate and theanine levels. Those findings demonstrate tissue-specific regulation of the ceRNA module, which is potentially modulated by elevated N levels and subject to feedback regulation via amino acid metabolites in tea roots.

Given the absence of a stable genetic transformation system for tea plants [46], we performed instantaneous silencing of miR1507c in tea roots and shoots, alongside transient co-expression assays of miR1507c, lncR12304.1, and *CsNADH-GOGAT* in tobacco. Considering the critical role of ethylamine in theanine biosynthesis, we strategically analyzed the ethylamine-generating substrate—alanine—in transformed tobacco. Due to the inherent detection limitations of ethylamine at very low physiological concentrations, its levels were difficult to detect unless artificially enhanced by overexpressing the *C. senensis* alanine decarboxylase gene (*CsAlaDC*) in tobacco [47]. Our results revealed a significant decrease in alanine content within the transiently transformed tobacco system. To some extent, this reduction also provides supportive evidence for the promotion of glutamate and theanine synthesis by the ceRNA module.

Considering the limitations of transient transformation, such as short-term effects or lack of stable inheritance, we had also attempted to perform genetic transformation assays to validate the regulatory relationships among miR1507c, *CsNADH-GOGAT*, and lncR12304.1. Our objectives include: (i) obtaining T2 generation homozygous transgenic *Arabidopsis* lines overexpressing *CsNADH-GOGAT*, miR1507c, and lncR12304.1 individually; and (ii) generating hybrid lines co-expressing *CsNADH-GOGAT* × miR1507c and *CsNADH-GOGAT* × miR1507c × lncR12304.1. Currently, T1 transgenic plants harboring *CsNADH-GOGAT*, miR1507c, or lncR12304.1 genes have been successfully obtained. However, screening of T2 generation *CsNADH-GOGAT* overexpression lines remains challenging. We speculate that the extended coding sequence length of *CsNADH-GOGAT* may contribute to reduced transformation efficiency. This technical hurdle is being actively addressed through protocol optimization.

**Figure 6:**
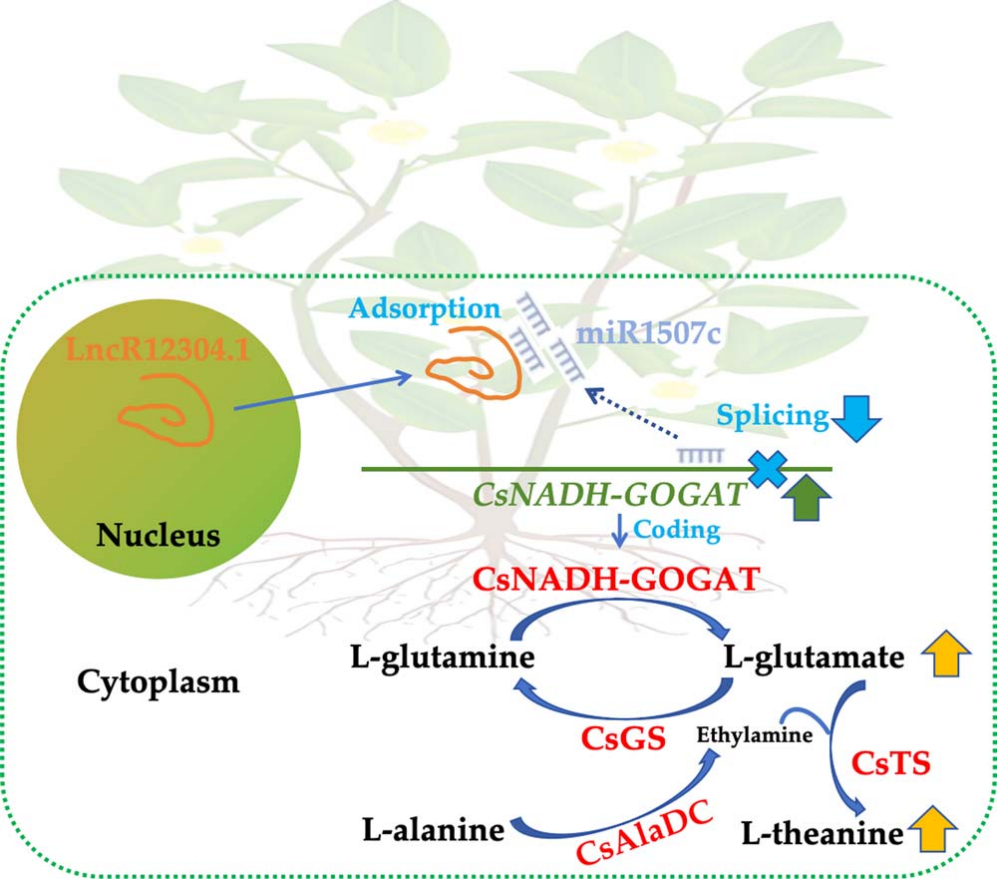
Proposed mode in which lncR12304.1 adsorbs miR1507c to weaken. *CsNADH-GOGAT* splicing and improve l-glutamate and l-theanine biosynthesis. Note: CsNADH-GOGAT, glutamate synthase; CsGS, glutamine synthase; CsTS, theanine synthase; CsAlaDC, alanine decarboxylase.

In conclusion, we propose a ceRNA regulatory module defined as lncR12304.1-miR1507c-*CsNADH-GOGAT* that regulates amino acids accumulation. Specifically, lncR12304.1 efficiently sequesters miR1507c, thereby attenuating miR1507c-mediated repression of *CsNADH-GOGAT* expression (Fig. 6). Based on transcript-level evidence supporting the involvement of this ceRNA module, our findings provide novel insights into the post-transcriptional regulatory mechanisms governing amino acid biosynthesis in tea plant. However, we acknowledge that direct quantification of CsNADH-GOGAT enzymatic activity and protein abundance would be essential to mechanistically link the observed changes in glutamate, theanine, and other amino acids to this regulatory module. Future studies will prioritize concurrent measurement of CsNADH-GOGAT activity and protein levels alongside transcript profiling to elucidate their relative contributions to amino acid flux. Furthermore, functional validation will probably be pursued through transient overexpression/knockdown of miR1507c in tea hairy root systems or stable transgenic lines targeting the ceRNA module components, in order to dissect their roles in the expression and activity of CsNADH-GOGAT protein as well as the regulation of amino acid metabolism.

## Materials and methods

### Nitrogen treatment and sampling of tea plant

Annual cutting ‘Shuchazao’ tea seedlings, which were planted under natural conditions, were chosen for nitrogen treatment. The urea (total nitrogen $\ge$ 46%, particle size: 2.00 to 4.75 mm) as nitrogen nutrition was formulated into solution with 1% (12.5 g/m^2^), 3% (37.5 g/m^2^) concentration. Then urea solution was filtered evenly into soil once every 20 days for two times totally. The control treatment of tea plant was irrigated equal amount of clean water. Tea shoot (bud and two leaves) and root were sampled after the second irrigation for 20 days, and frozen by liquid nitrogen, finally stored in −80°C for subsequent use.

### LncRNA sequencing and analysis

Using tea shoots and roots as materials, the total RNA was extracted using the CTAB method. The lncRNA library was constructed using the Ribo-off rRNA depletion kit (Plant, Vazyme #N409) and VAHTS Universal V6 RNA-seq Library Prep Kit for Illumina (Vazyme #NR604). Then the sequencing was performed on the Illumina NovaSeq X Plus by Gene Denovo Biotechnology Co., Ltd (Guangzhou, China). The high-quality clean reads were obtained by Fastp (v0.18.0) filter, and then the rRNA-mapped reads were removed. The paired-end clean reads were mapped to the ‘Shuchazao2’ reference genome (http://tpia.teaplants.cn) using HISAT2 (v2.1.0), and all transcripts were reconstructed by Stringtie (v1.3.4) together with HISAT2.

The reliable novel transcripts were identified using Cuffcompare and used to assess the protein-coding potential through software CNCI (v2), CPC (v0.9-r2), and FEELNC (v0.2). The intersection of both non-protein-coding potential results was chosen as long non-coding RNAs (lncRNAs). Using RSEM software, TPM values were calculated to quantify LncRNAs expression abundance. Based on the lncRNA levels in each sample, correlation between samples was analyzed by principal component analysis (PCA) and Pearson coefficient. LncRNAs differential expression analysis was performed by DESeq2 with the parameter of FDR < 0.05 and |log_2_FC| > 2. Utilizing the Patmatch (v1.2) software, candidate lncRNAs that bind to the target miRNA were screened from the entire lncRNAs dataset. Subsequently, those with a binding energy of less than −23.5 kcal/mol and a minimum free energy (MFE) ratio greater than 70% were chosen for further experimental validation.

### Dual-luciferase reporter assay

The precursor sequence of miR1507c was inserted into pGreenII-SK-62 vector, about 200 bp of lncR12304.1 and *CsNADH-GOGAT* sequence near the predicted binding site with miR1507c were inserted into pGreenII0800-LUC vector. Then reconstructed plasmids were transformed into *Agrobacterium* for culturing 2 to 3 days. Select monoclonal *Agrobacterium* containing the target vector and expand the culture in LB liquid medium containing 10 ml antibiotic (K + Rif^+^) for approximately 36 to 48 h. Mix two *Agrobacterium* solutions with equal volume and then inject into leaves of tobacco, which were sampled after culturing at around 28°C for 48 h.

A portion of the leaves were injected with 1 × D-fluorescein (Biotium, PI-10100) for observing whether there was fluorescence by Tanon 5200 chemiluminescence apparatus. The other portion of tobacco leaves without injected with D-fluorescein were used to detect Firefly luciferase (LUC) and Renilla luciferase (REN) genes activities by LUC reporter gene assay kit (Sangon, E607406). Using REN fluorescence value as an internal reference, the value of LUC/REN presented the eternal result.

To further identify the interaction between miR1507c and *CsNADH-GOGAT*, three bases ‘CTA’ were inserted to *CsNADH-GOGAT* target sequence correspondingly to the middle of the miRNA 10th to 11th base [39]. It caused that the miRNA could not normally cleave *CsNADH-GOGAT*m3, which was similarly to construct recombinant plasmid with pGreenII0800-LUC. Then three group were transformed into tobacco leaves with 1:1 bacterial solution, including group A, pGreenII0800-LUC-*CsNADH-GOGAT* & pGreenII-SK-62-none; B, pGreenII0800-LUC-*CsNADH-GOGAT*m3 & pGreenII-SK-62-miR1507c; C, pGreenII0800-LUC-*CsNADH-GOGAT* & pGreenII-SK-62-miRNA. After injection for 1, 2, 4, 7 days, the D-Luciferin potassium salt was applied to the leaves underside for fluorescence photography. And the LUC fluorescence value were detected using REN fluorescence value as control at the second and seventh days. Meanwhile the expression levels of miRNA, *CsNADH-GOGAT*, and two tobacco *NtNADH-GOGAT* (LOC107809098, LOC107822887) (Fig. S2) were detected by RT-qPCR.

### 5′RLM-race

The total RNA were extracted using TRIzol method (Sangon, B511321), and RNA pretreatment and addition of artificial adapters referenced to the Gene Racer™ Kit manual (Invitrogen, L150001). RNA was reversed to the first cDNA with 5′RACE reverse primers. Using the diluted cDNA, gene special primer 1 (GPS1) and RLM-RACE 5′Primer, the first PCR was performed; using the first diluted PCR product, GPS2 and RLM-RACE 5′Nested Primer, the second round PCR was completed. Gel extracted nested PCR product (Sangon, B518131) was transformed to *E. coli* competent cells SK2301, and amplified with universal primers of pMD18-T vector. According to the sequencing result of colony PCR, the cleavage site between miRNA and target mRNA was identified. Those primers sequences for 5′RLM-RACE were shown in Table S3.

### RT-qPCR analysis

Using two-step RT-qPCR method, the total RNA was extracted (TIANGEN, DP441) form tea plant or tobacco leaves and reverse transcribed into cDNA with reverse transcriptase (Thermo Scientific, EP0743). The expression levels of miR1507c, lncR12304.1, and mRNA were performed with 2× SG Fast qPCR Master Mix (BBI, B639271) on qPCR instrument (Roche, LightCycler480 II) and calculated by 2^−ΔΔCt^ method. The PCR procedure was as follow: 95°C for 3 min followed by 45 cycles of 95°C for 15 s and then 60°C for 30 s. The primers sequences for RT-qPCR were shown in Table S4.

### Fish

Fluorescence in situ hybridization (FISH) was performed using specific probes as follow: miR1507c probe, 5′-cy3-AGAUGAUGUUUGGAAUGAGG-3′; lncR12304.1 probe, 5′-FITC-GAAACAACUAACAAACCAUGCCUCACUGAUCUUGAC-3′. The FISH experiment was performed refer to previously report [48]. The tender tea roots were fixed in 4% paraformaldehyde for 12 h. After dehydrating, dissecting, and deparaffinizing the samples, root were permeabilized with proteinase K (20 μg/ml) at 37°C for 30 min. Slides were then rinsed three times in phosphate-buffered saline (PBS) for 5 min. After prehybridization at 37°C for 1 h, tea roots and RNA probes (8 ng/ml) were incubated overnight at 37°C. Slides were washed at 37°C with 2× standard sodium citrate (SSC) for 10 min and then 1× SSC for 2 × 5 min, finally at 25°C with 0.5× SSC for 10 min. Nuclei were counterstained with DAPI solution (2 μg/ml) for 8 min in the dark, and added anti-fluorescence quenching mounting medium after rinse. The images were collected using an upright fluorescence microscope (BX63, Olympus).

### RNA pull down

The positive probe was designed on the basis of miR1507c, synthesized, and labeled with biotin. Meanwhile, a negative probe was designed as control. Then, qualified probes without RNA was obtained through isolating and purifying. Tea leaves were washed using PBS without RNA and lysed with cell lysis buffer. Supernatant was collected for reserving after centrifuging at 14 000 rpm for 15 min. The streptavidin beads was prepared by washing twice with wash buffer, and added biotinylated probe for incubating at room temperature with shaking for 1 h, then washed twice again. Next, the cell lysate was added for incubating overnight at 4°C, then washed five times. Using 50 μl elution buffer, the pull down RNA was resuspended and incubated at 100°C with shaking for 10 min. The RNA sample was collected and reverse transcribed into cDNA for RT-qPCR. Two primer pairs flanking the miRNA binding site (GGATGATGT/TGGAATGA) were designed to amplify partial lncRNA sequences. The miR1507c probes and lncR12304.1, *CsNADH-GOGAT* primers for qPCR were shown in Table S5. Both the RNA pull-down process and the corresponding qPCR were conducted as biological replicates.

### MiRNA suppression

The corresponding miR1507c Antagomir, which as combination inhibitor of miRNA and target gene, were synthesized by Mixsungen Biotechnology Co., Ltd. Isolated tea shoot (one bud and two leaves) and root were soaked in 1 ml of 20 μM Antagomir solution, and the Agomir were used as the control (Fig. S3). The samples were harvested at 10 h, then the levels of miR1507c, lncR12304.1, and *CsNADH-GOGAT* were identified by RT-qPCR, the contents of glutamate and theanine were measured by LC–MS.

### Transient tobacco transformation

The *CsNADH-GOGAT* CDS was amplified in three segments from tea leaves and successively ligated into the pCambia2301 vector backbone using KpnI restriction sites. Concurrently, the precursor and mature sequences of miR1507c and lncR12304.1 were cloned and inserted into pCambia3301-KY and pCambia1301-JC, respectively (Table S6). Took the wild type (WT) tobacco as control, three transformation groups were designed: (i) single *CsNADH-GOGAT* transformation, (ii) miR1507c + *CsNADH-GOGAT* co-transformation, and (iii) lncR12304.1 + miR1507c + *CsNADH-GOGAT* co-transformation into tobacco leaves. Tea shoots and roots were harvested at the first and fourth days post-transformation (dpt) for quantification of gene abundance via RT-qPCR and l-glutamate levels via LC–MS.

### LC–MS

The samples of tea or tobacco leaves were grinded and crushed in liquid nitrogen and weighed accurately. Using 4 ml of 20% ethanol solution containing 0.001 M HCl, the amino acids were extracted under a low-temperature water bath for 30 min, followed by centrifugation at 12 000 *g* at 4°C for 5 min. The supernatant were collected and added 4 ml of extraction buffer to the precipitate for re-extraction. Two supernatants were combined and diluted to 10 ml. After mix thoroughly, the amino acid crude extract were filtered through 0.22 μm membrane for detection. The LC condition were as follows: chromatographic column, Agilent InfinityLab Poroshell 120 HILIC-Z (2.1 mm × 100 mm, 2.7 μm); column temperature, 35°C; mobile phase: A, 10 mM ammonium formate (pH = 3); B, 90% acetonitrile (containing 10 mM ammonium formate, pH = 3); flow rate, 0.3 ml/min; injection volume, 1 μl; elution gradient (A phase): 0–2 min, 5%; 2–4 min, 5% to 20%; 4–6 min, 20% to 45%; 6–12 min, 45%, 12 to 12.1 min, 45% to 5%; 12.1–15 min, 5%. The MS parameters were as follows: Capillary (kV): 2.5, Source offset (V): 50, Source temperature (°C): 150, Desolvation temperature (°C): 500, Cone gas flow (L/Hr): 150, Desolvation gas flow: 1000.

### Statistics analysis

All experiments were performed in triplicates, including dual-luciferase reporter assay, RNA pull down, FISH, RT-qPCR and LC–MS, and the data generated by three experimental repeats were organized and statistically analyzed using Excel 2010 and SPSS software. The statistical significance between groups was analyzed using Student’s *t*-test. All the bar plot in this paper were completed by the Chiplot online tool (https://www.chiplot.online/).

## Supplementary Material

Web_Material_uhag014

## Data Availability

All relevant data in this study are provided in the article and its supplementary files. The raw data of lncRNA in this study had been submitted to NCBI Sequence Read Archive (ID: PRJNA1304500).
