# Supplementary material for: LncR12304.1-miR1507c-CsNADH-GOGAT ceRNA module regulates amino acid biosynthesis in tea plant (Camellia sinensis)
Source: Hortic Res. 2026 Jan 13;13(4):uhag014. doi: 10.1093/hr/uhag014 (PMC13095357; doi:10.1093/hr/uhag014)
Supplement: Web_Material_uhag014 [file web_material_uhag014.zip › Figure S2.docx]

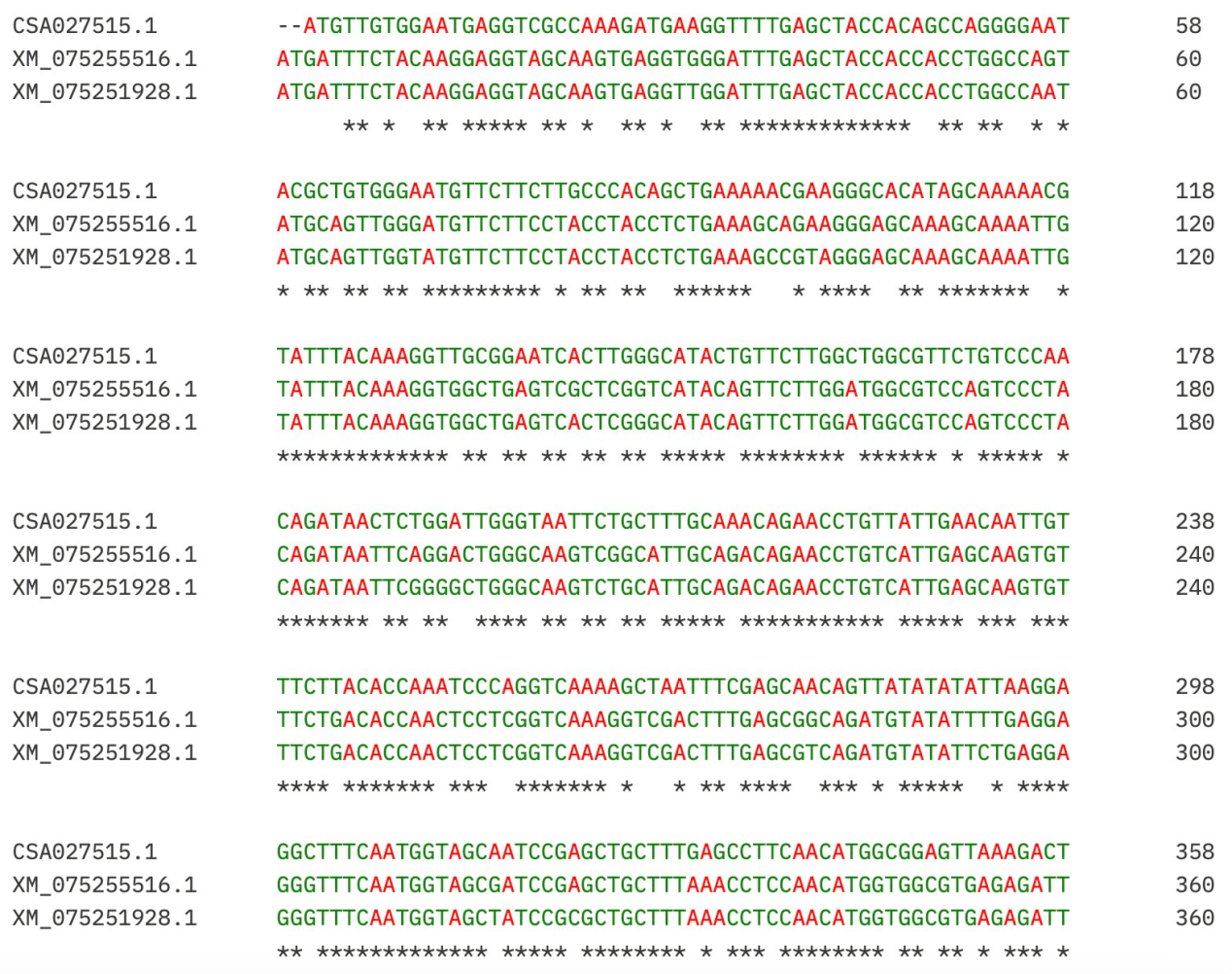


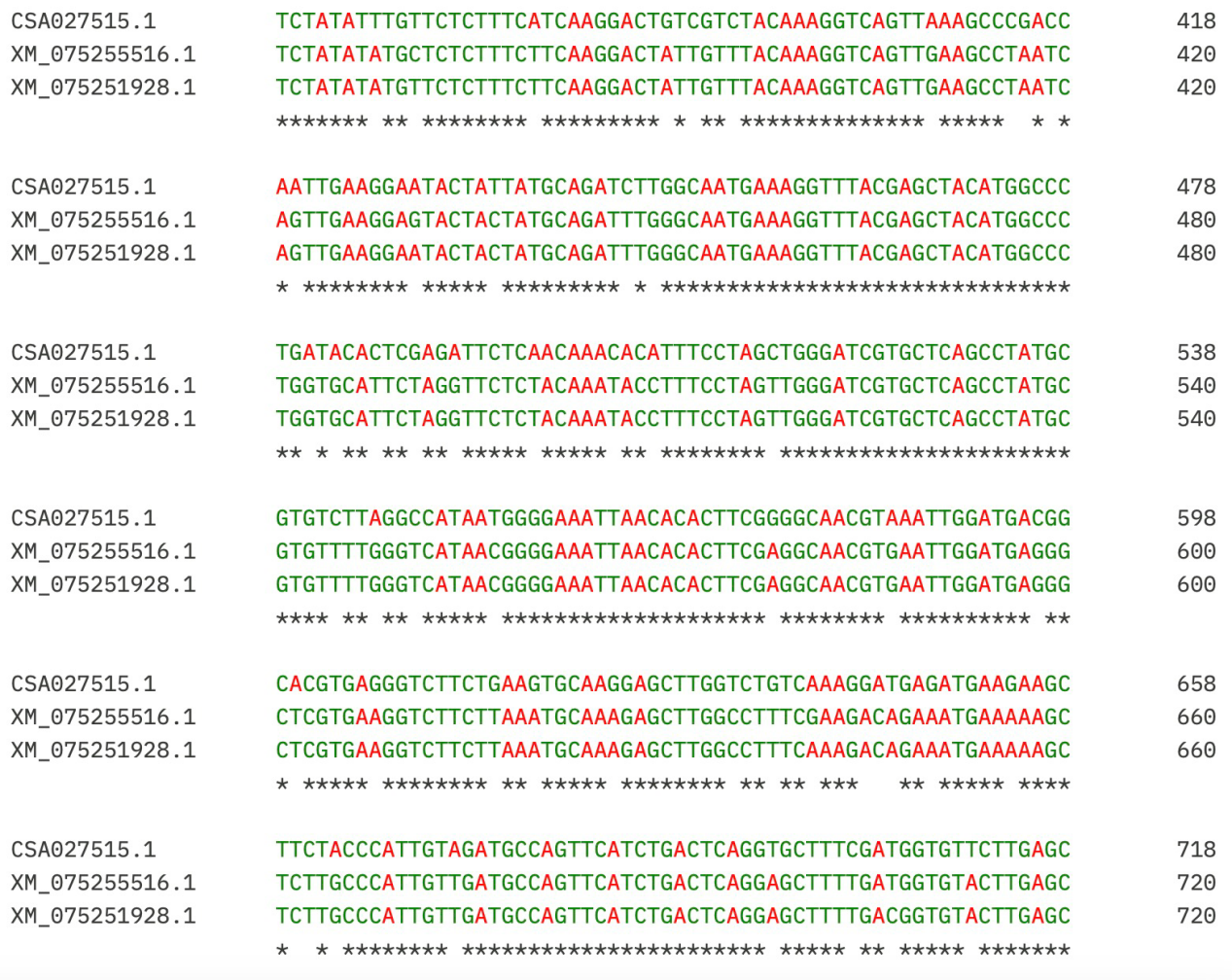


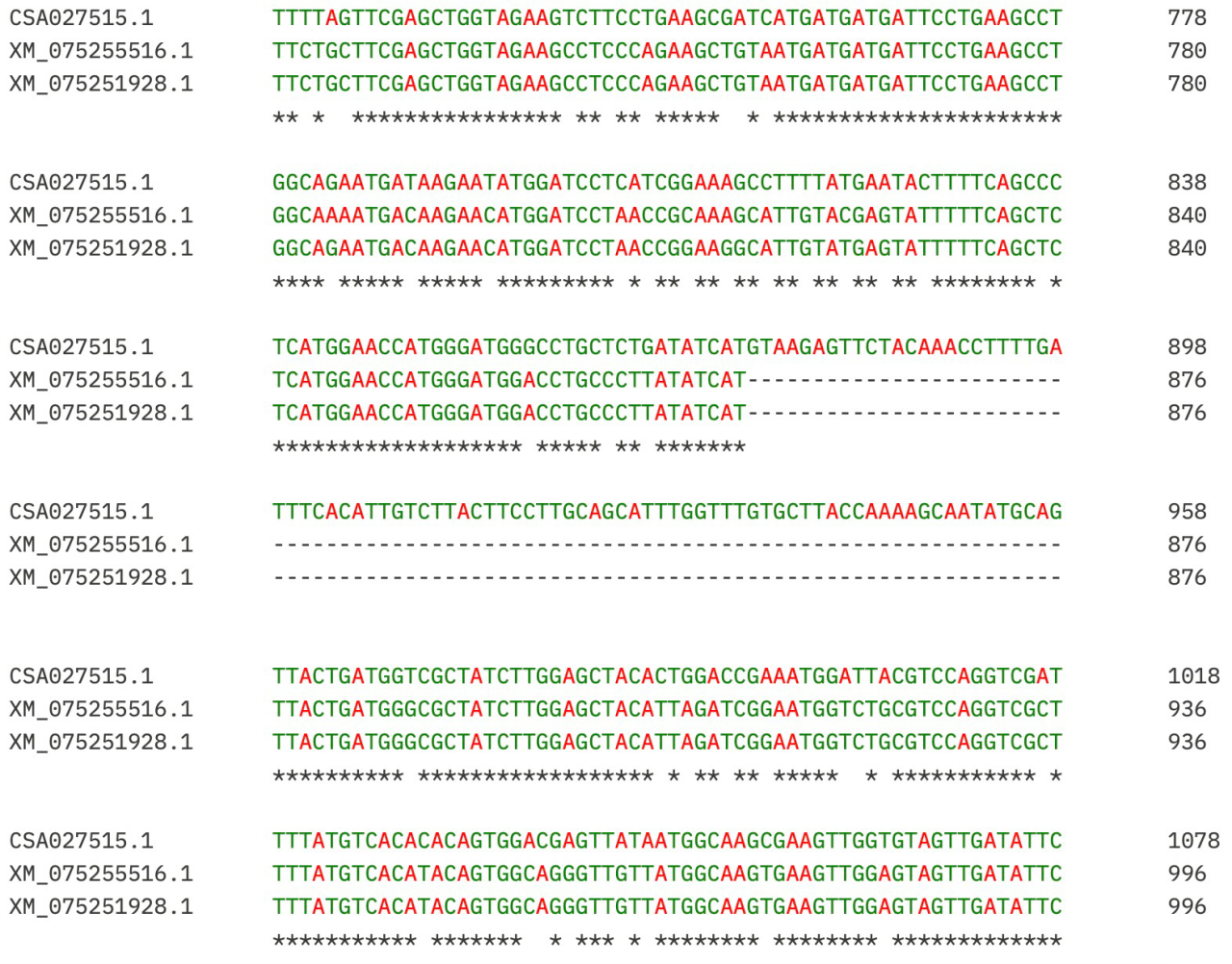


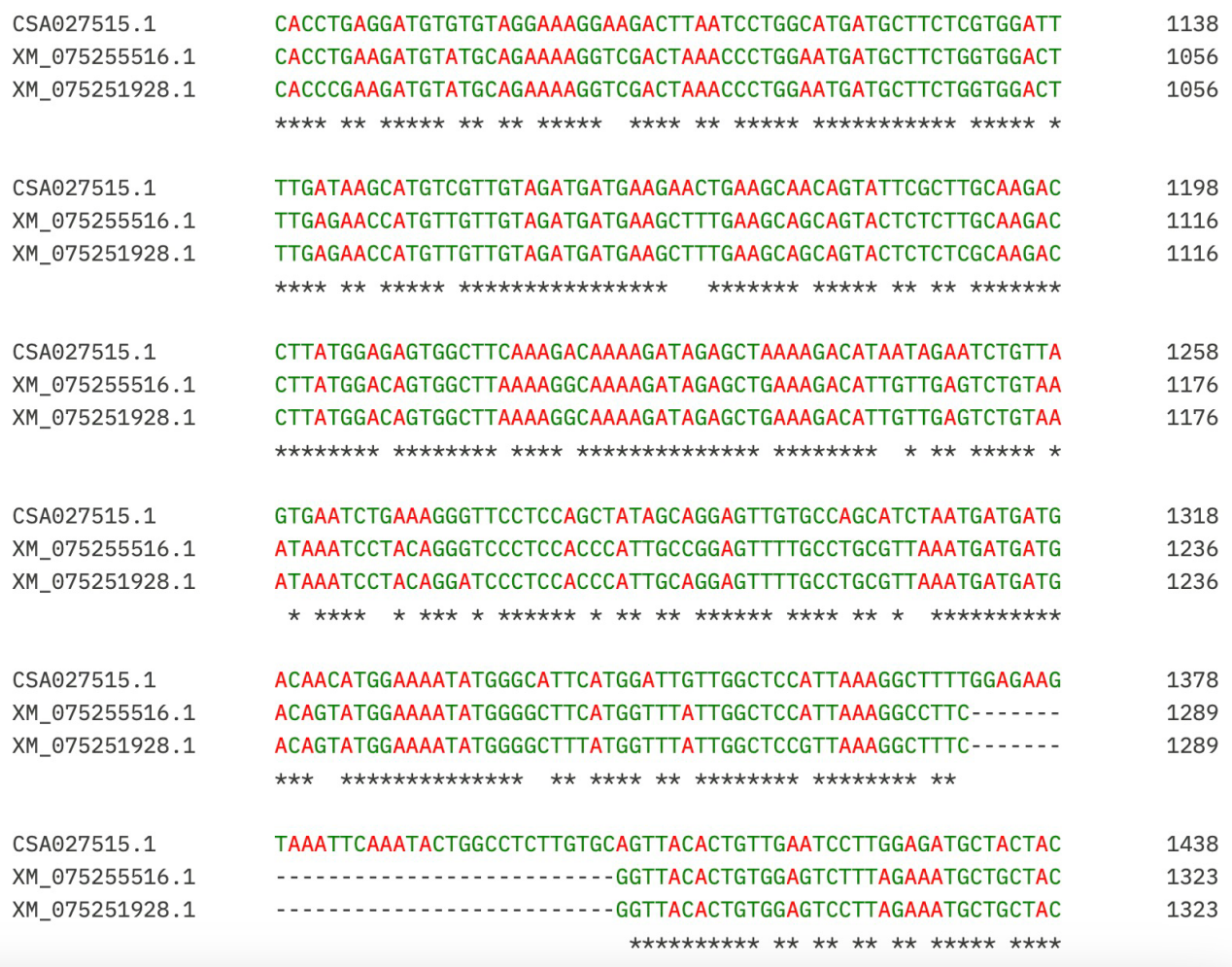


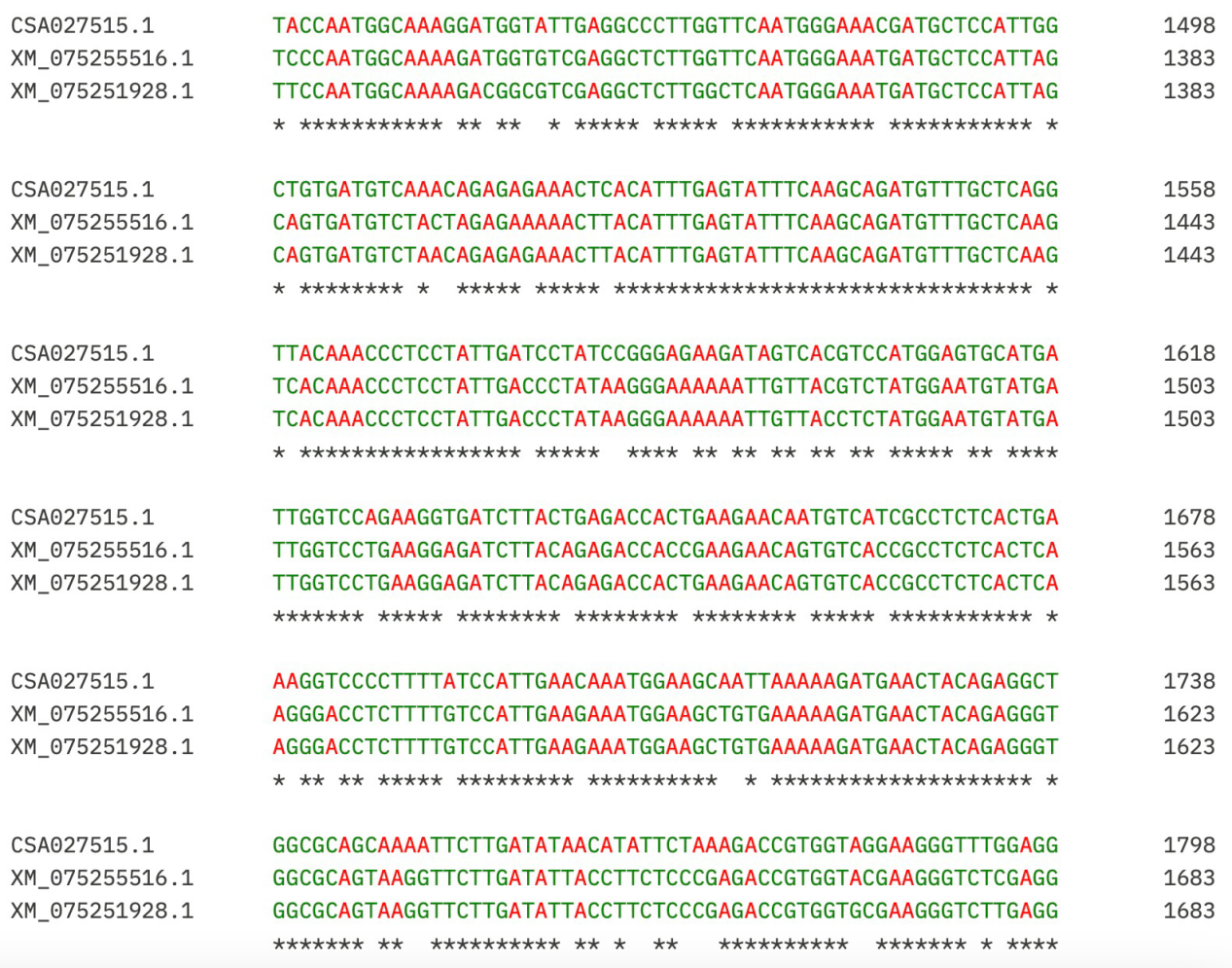


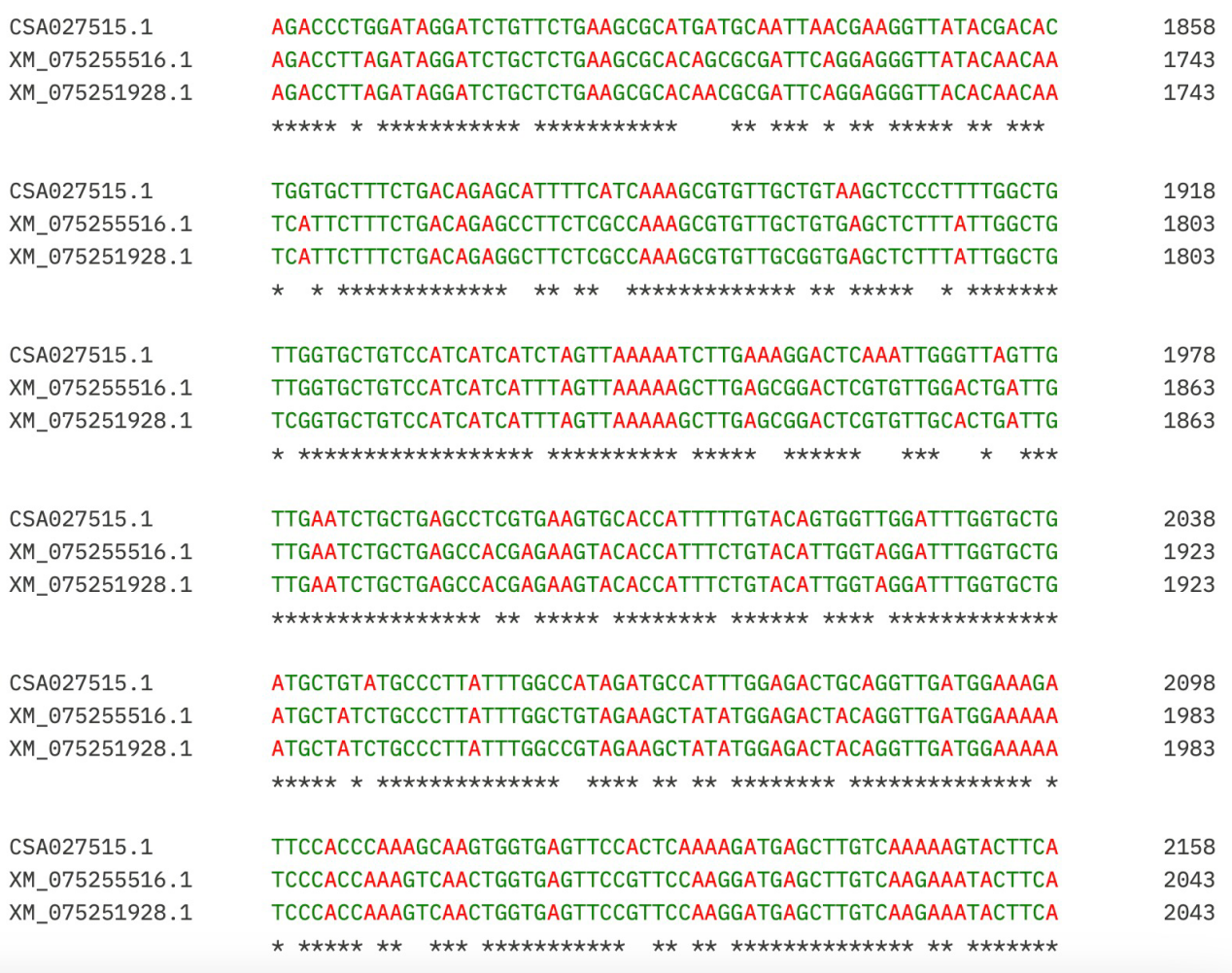


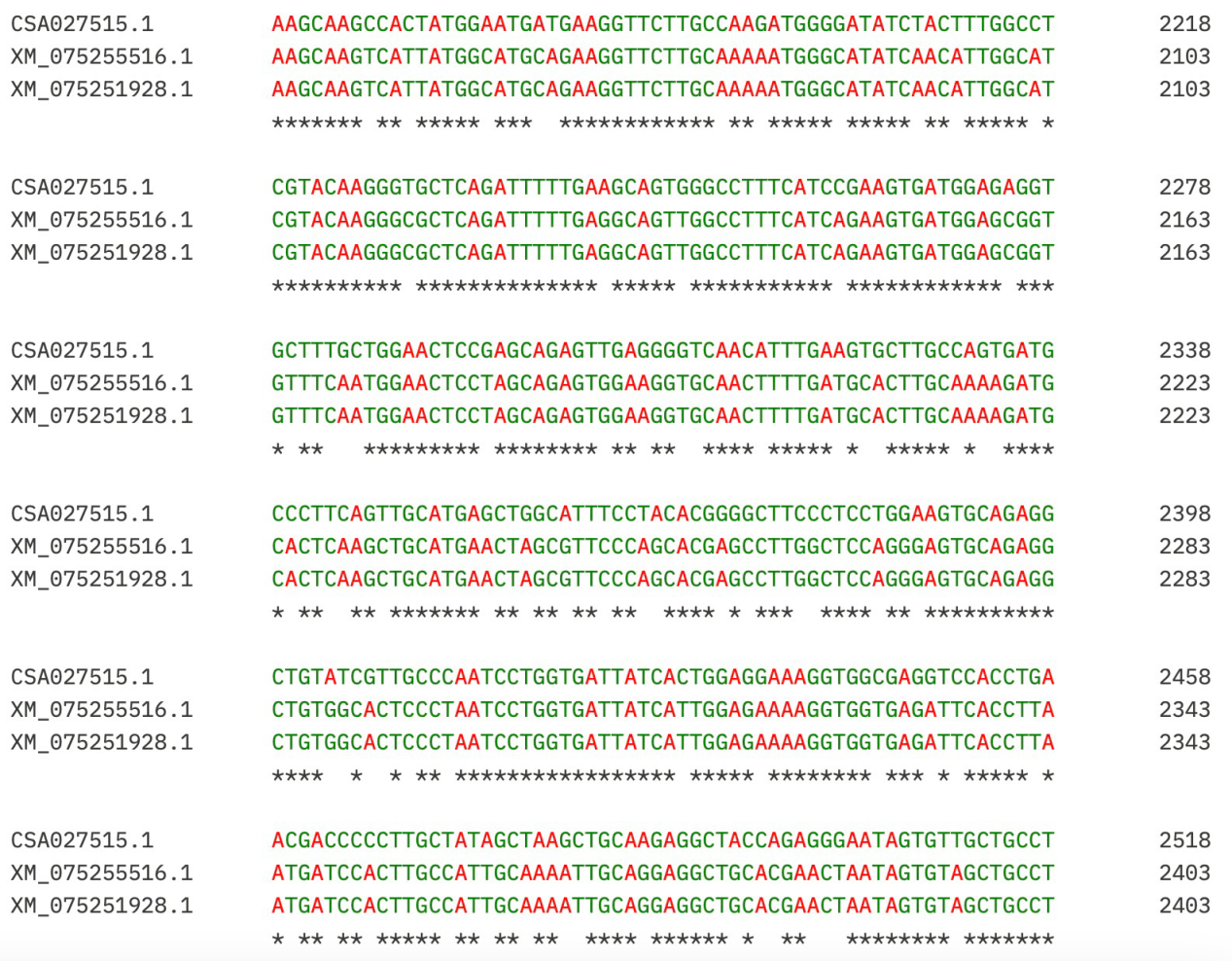


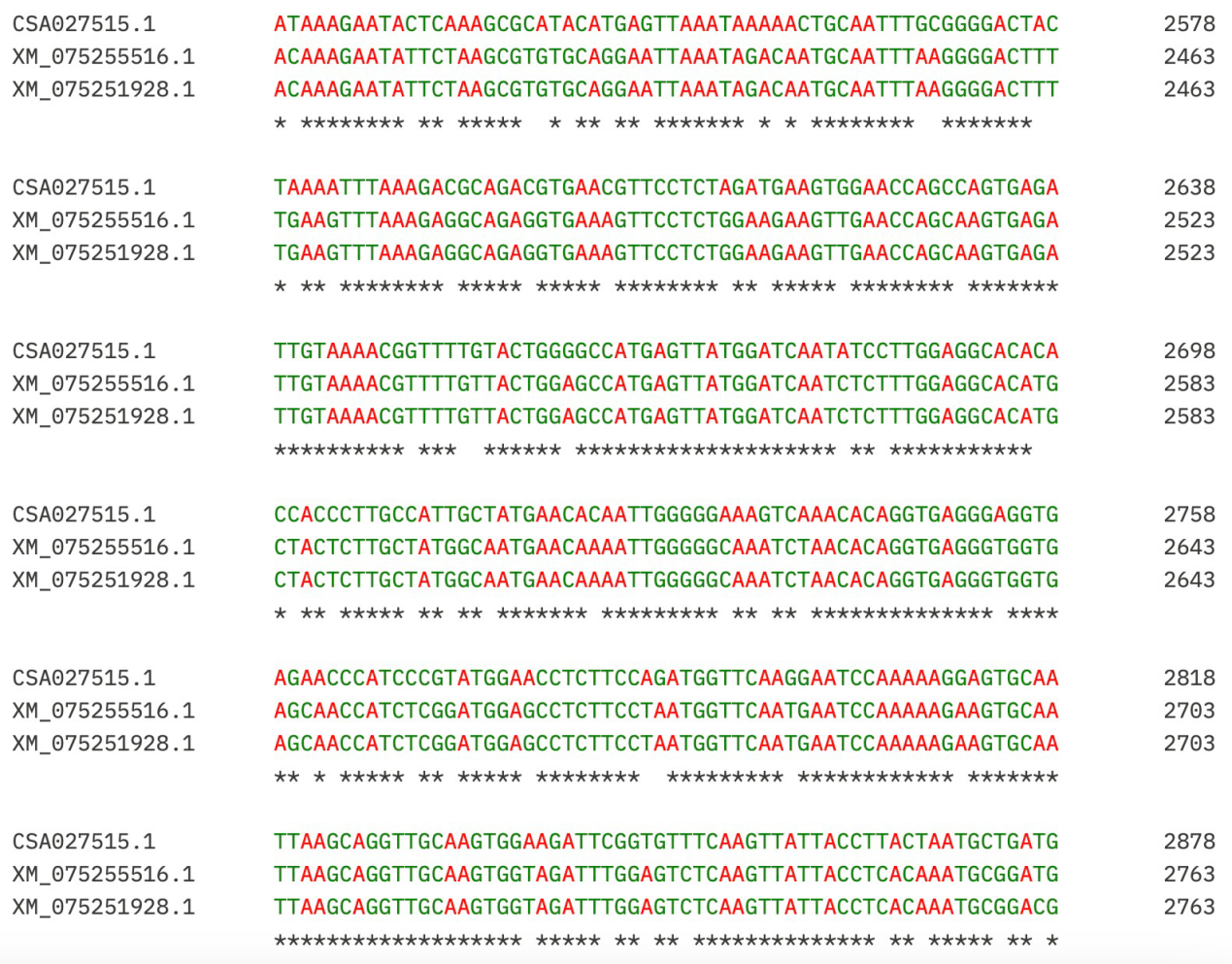


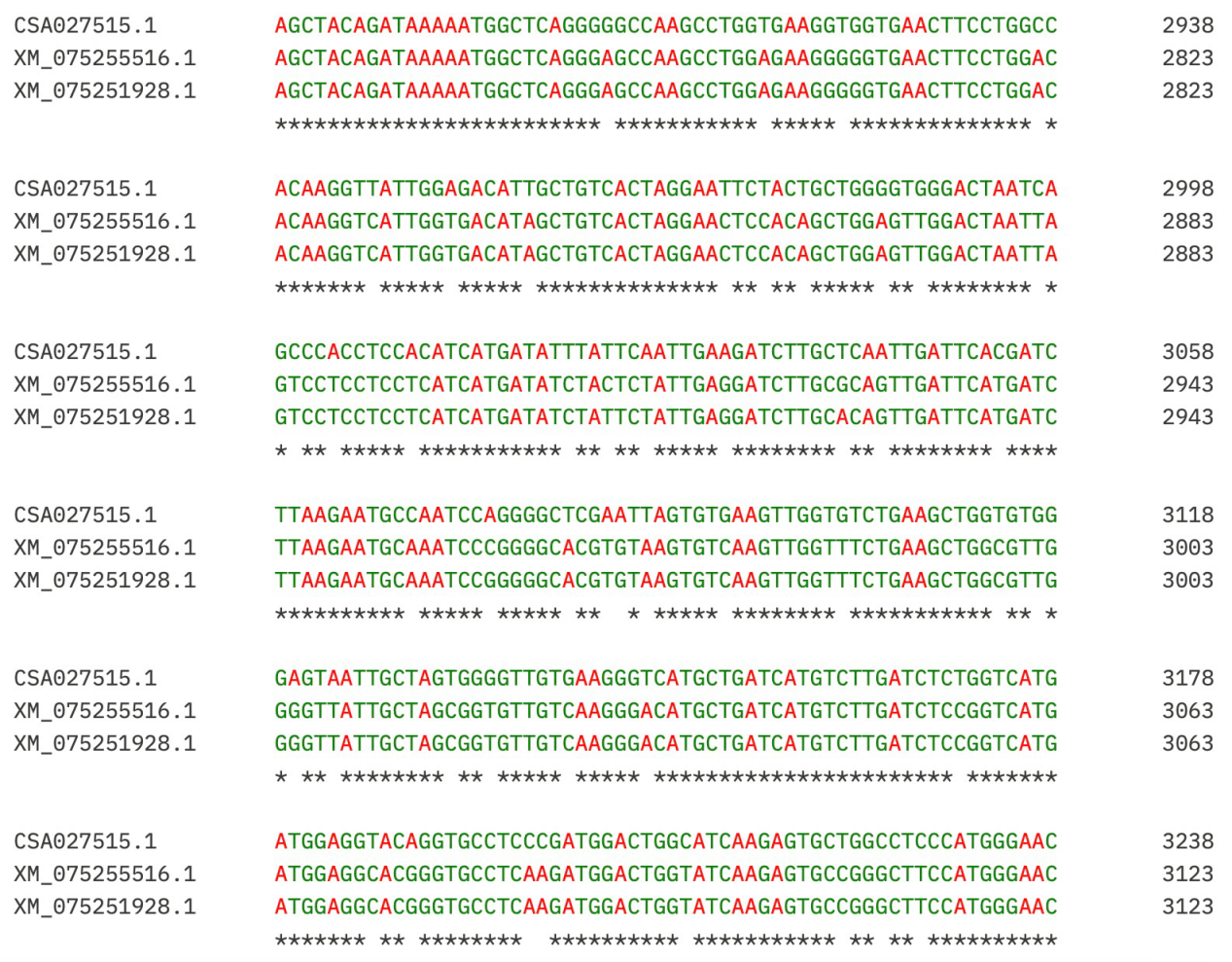


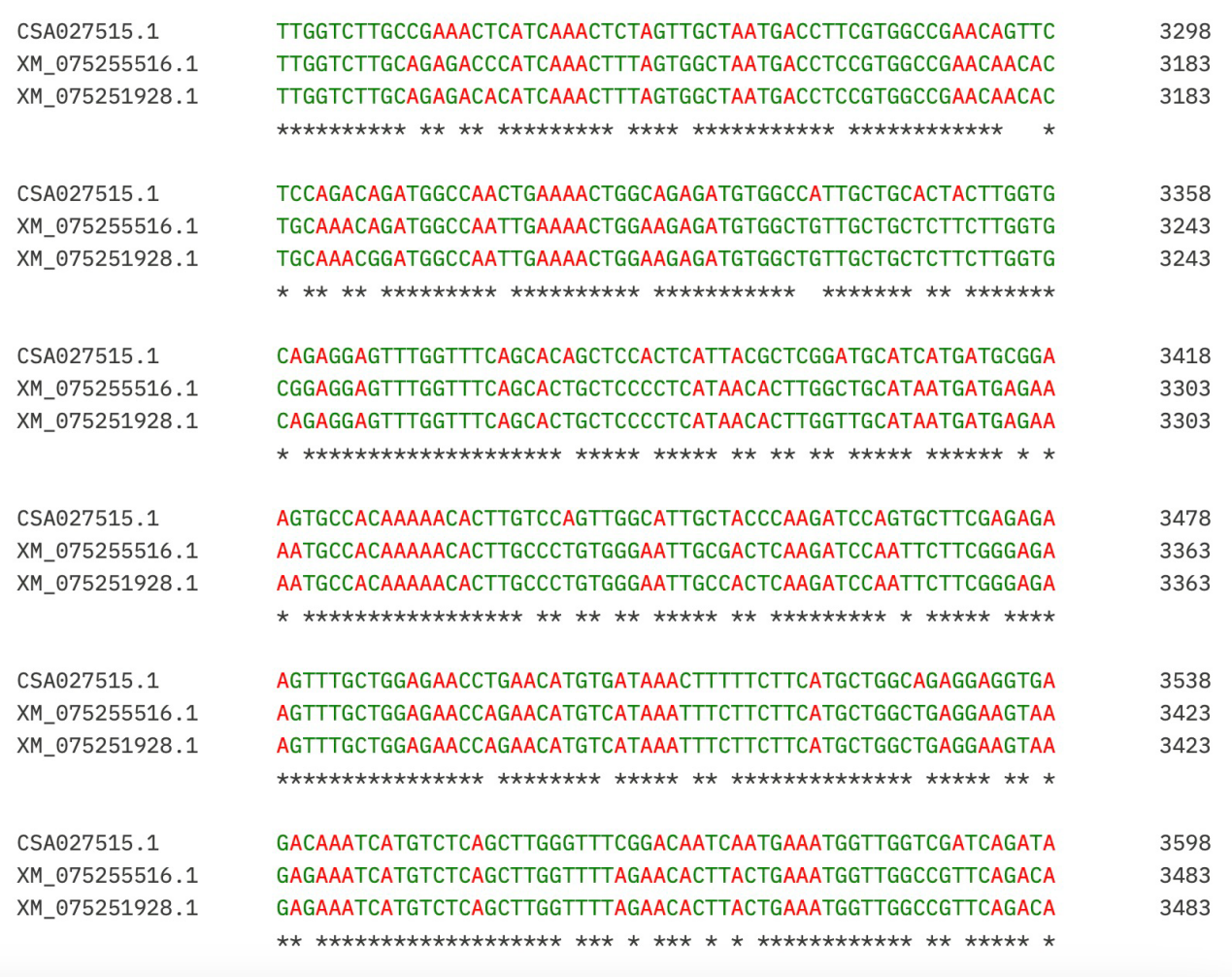


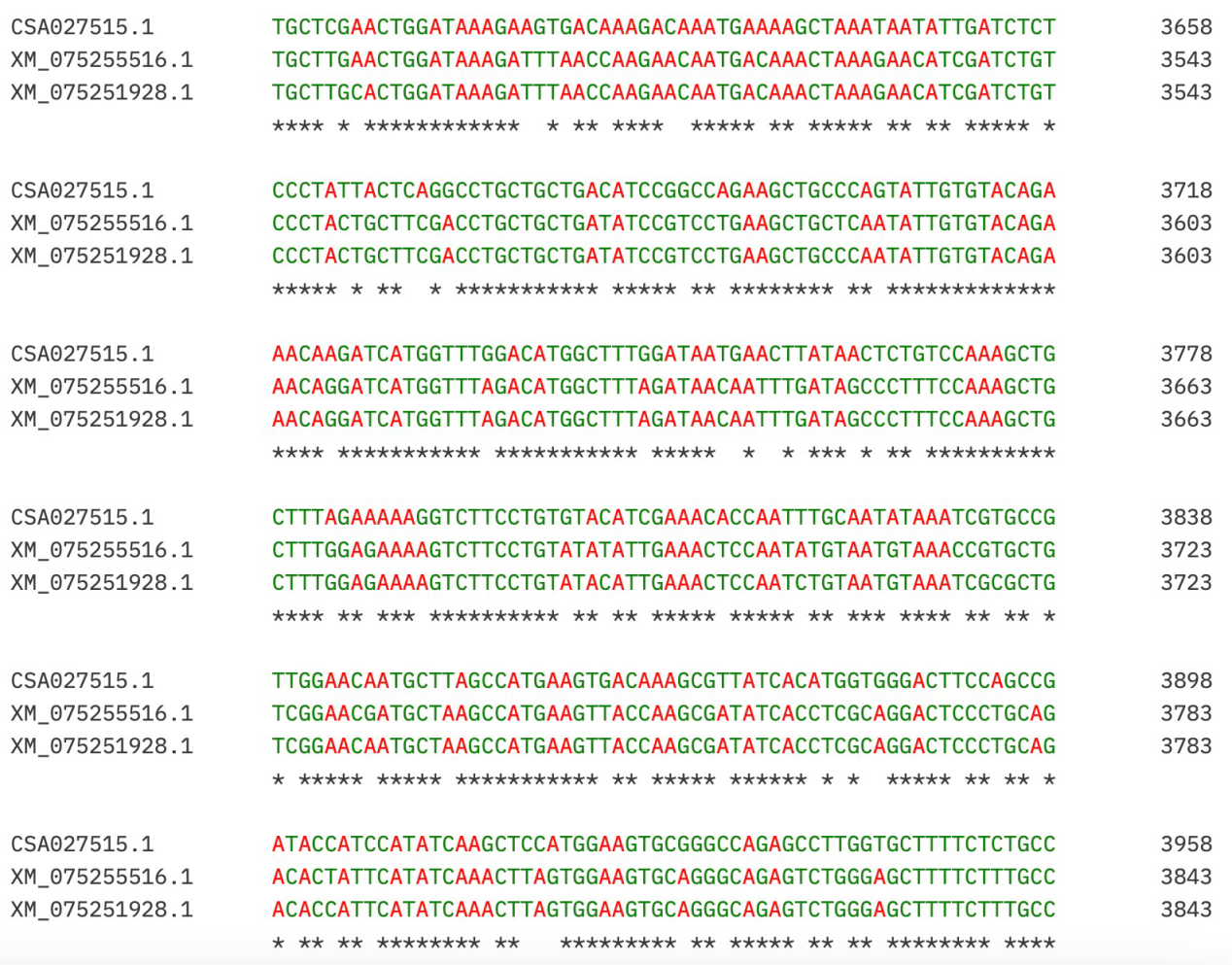


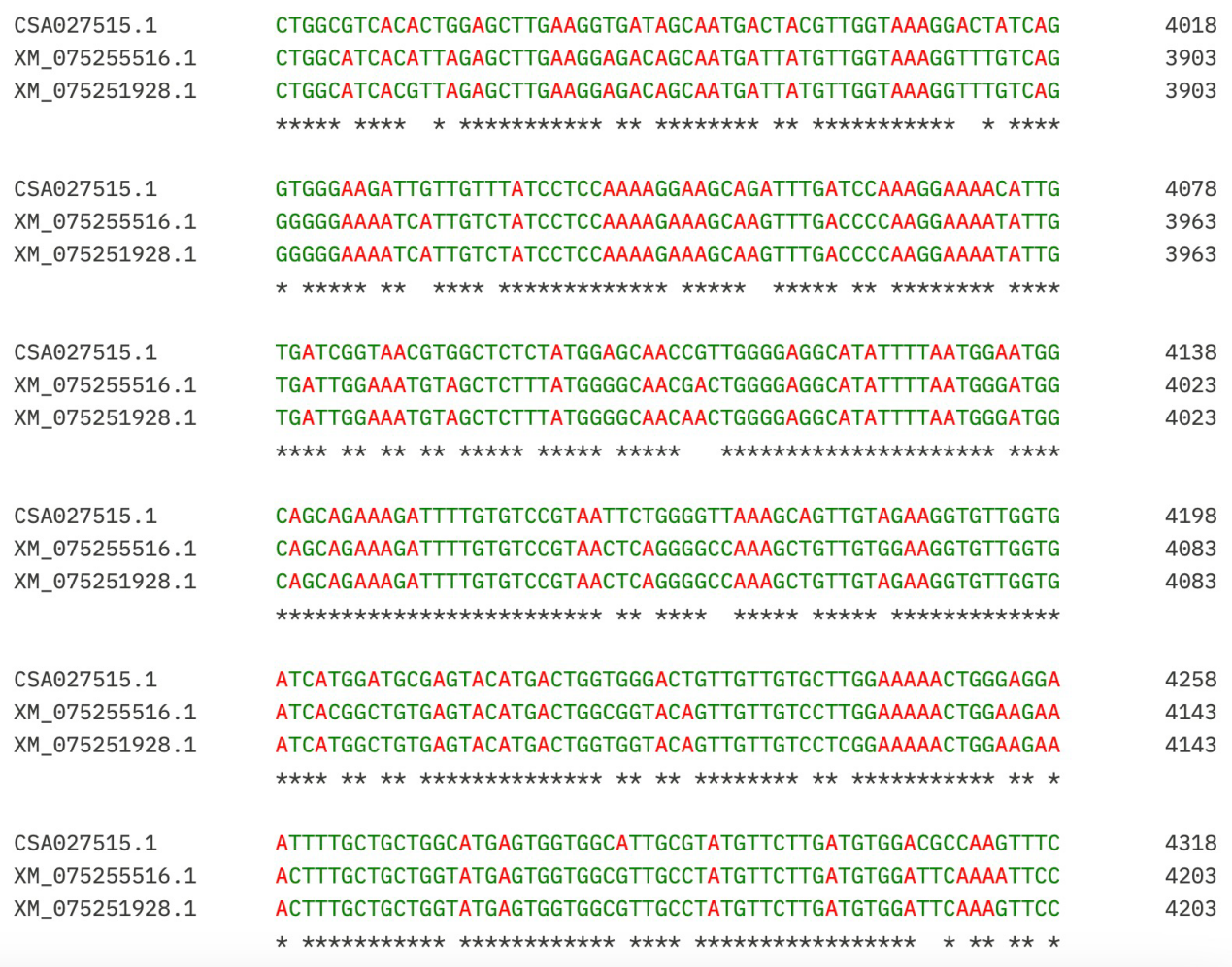


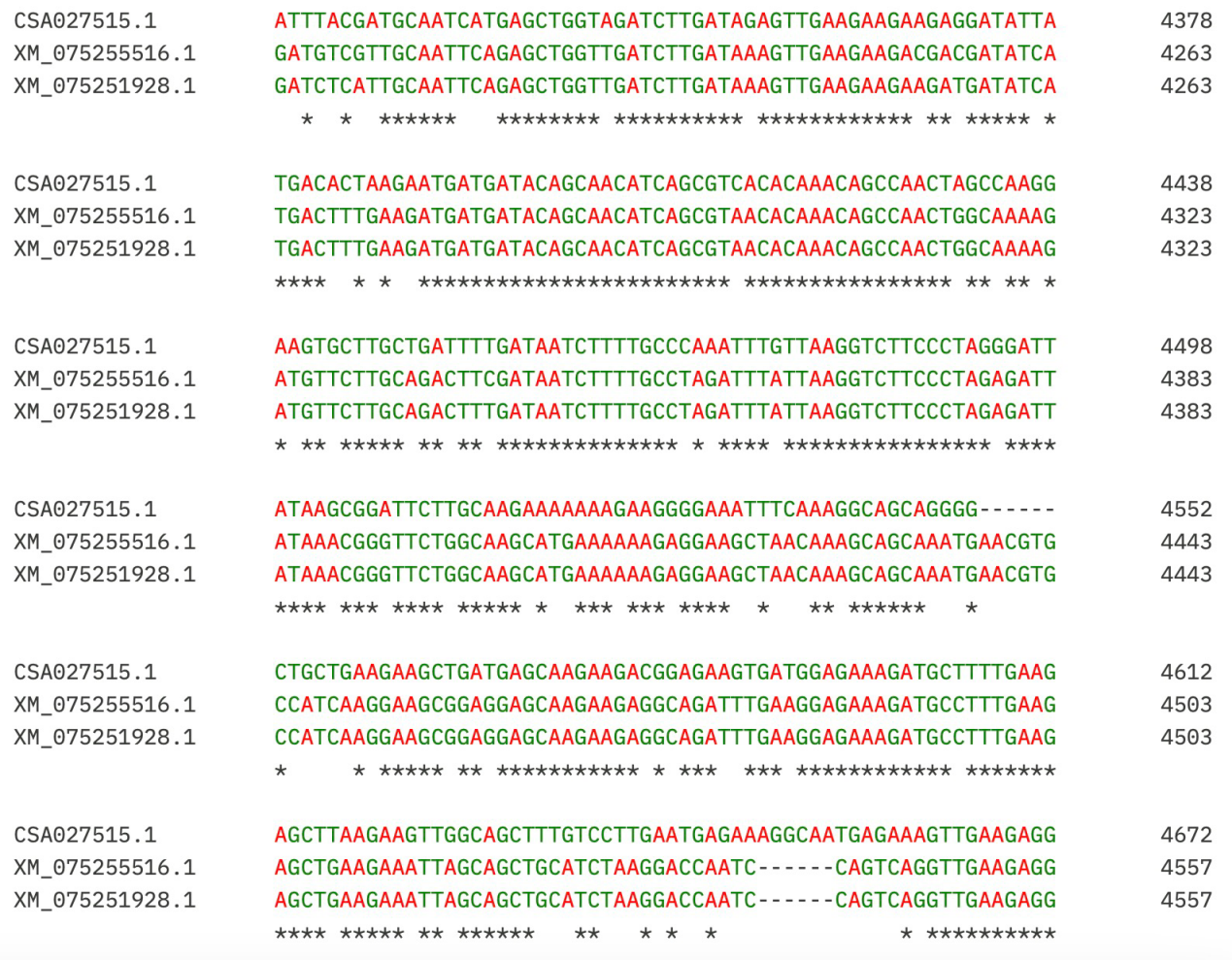


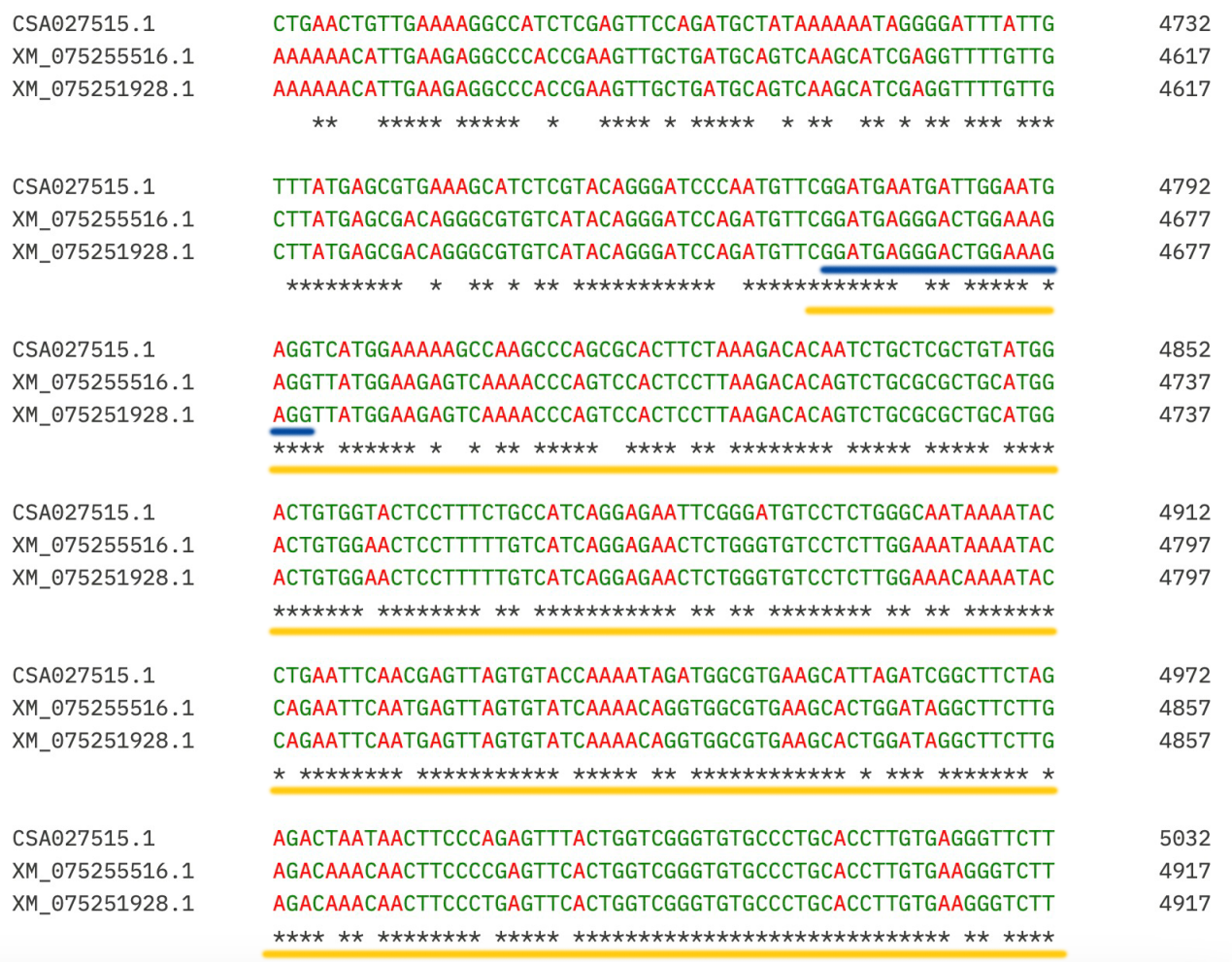


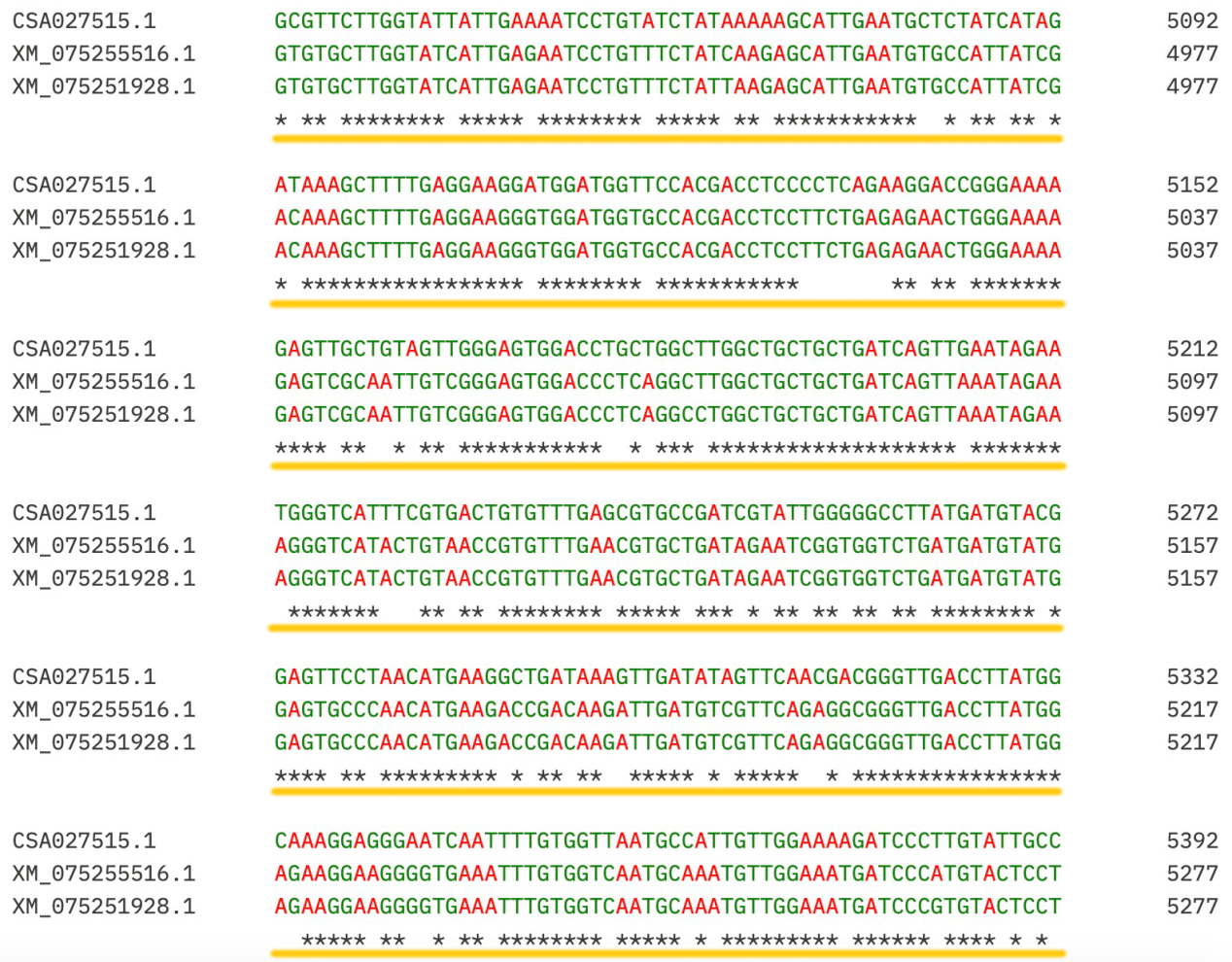


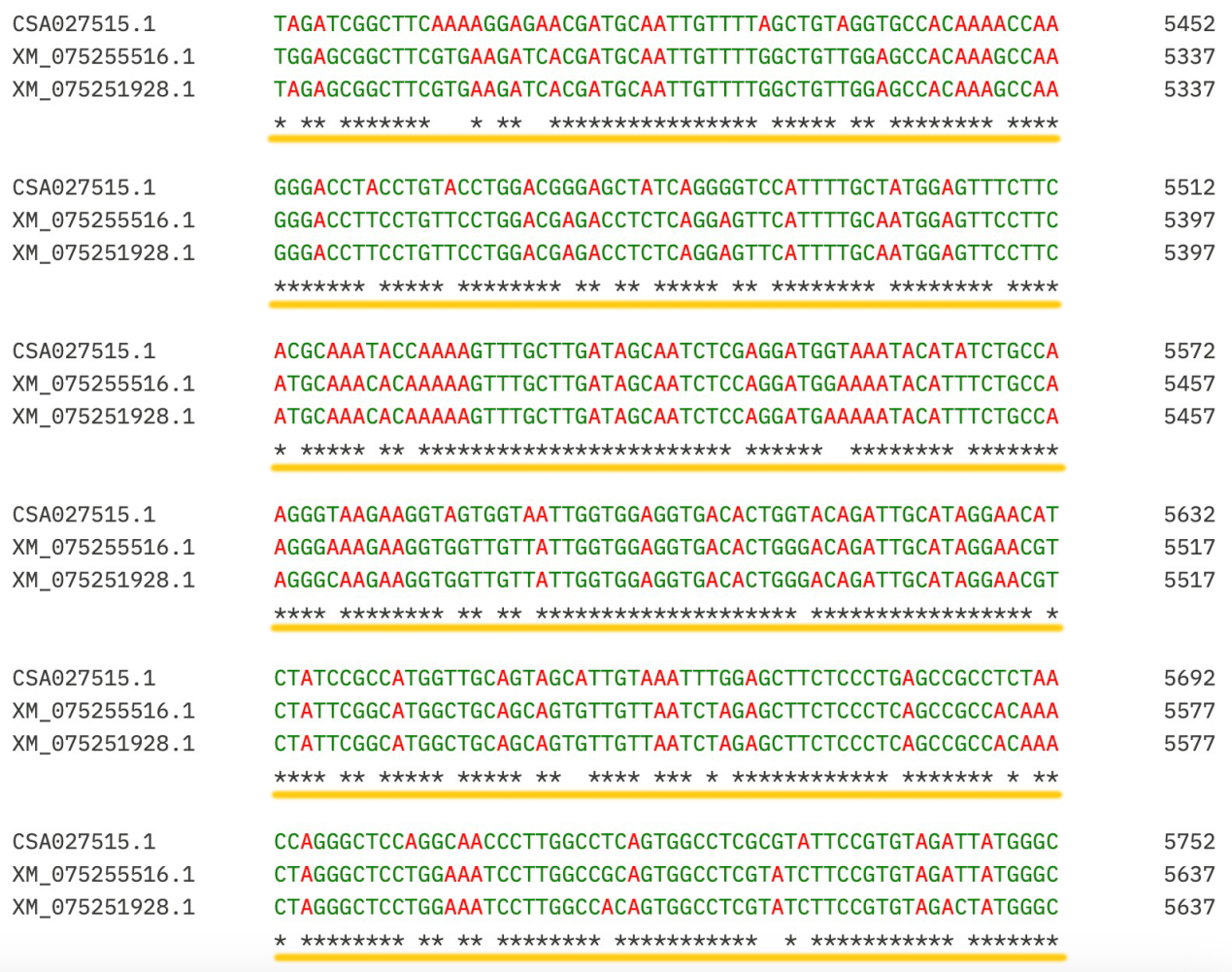


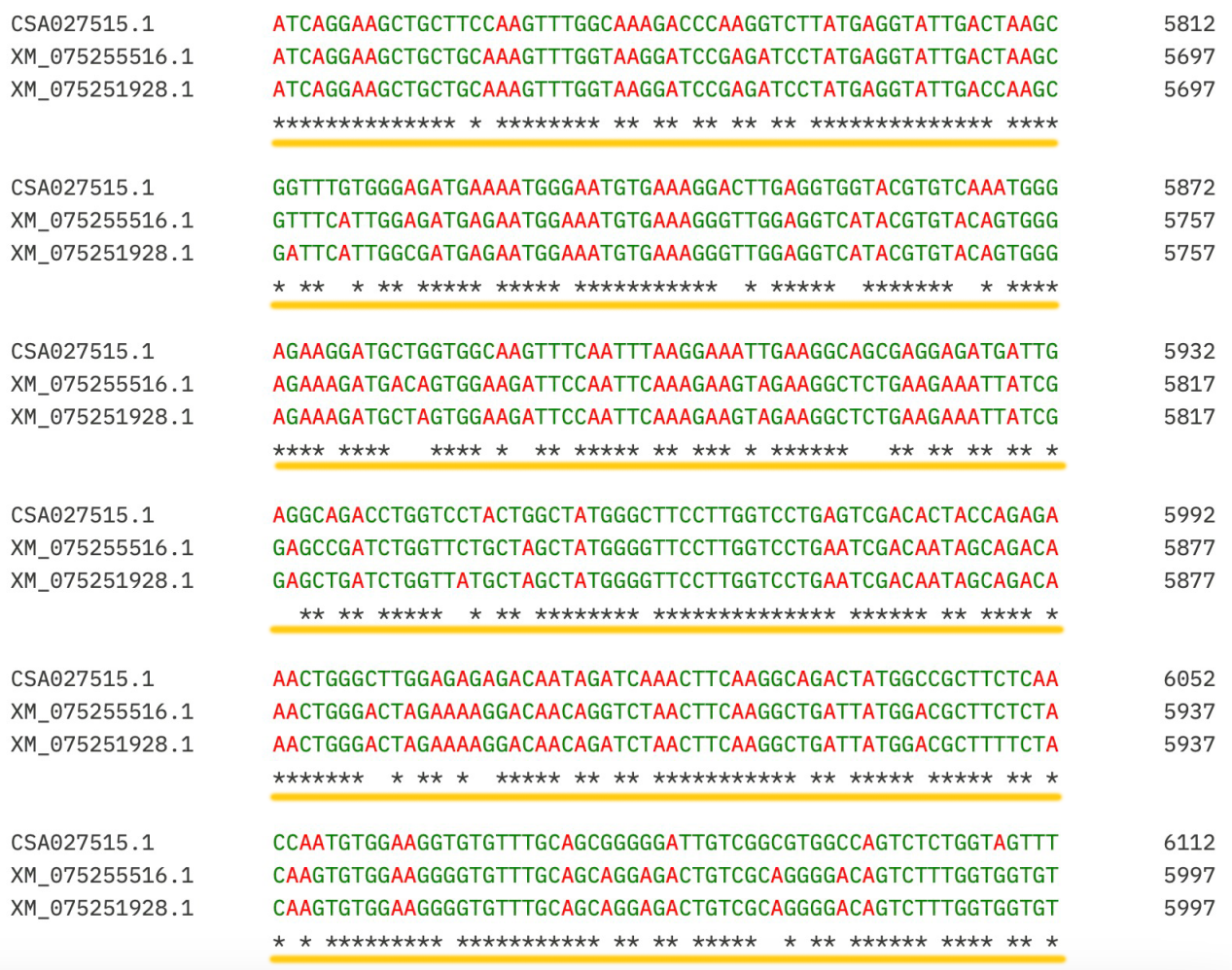


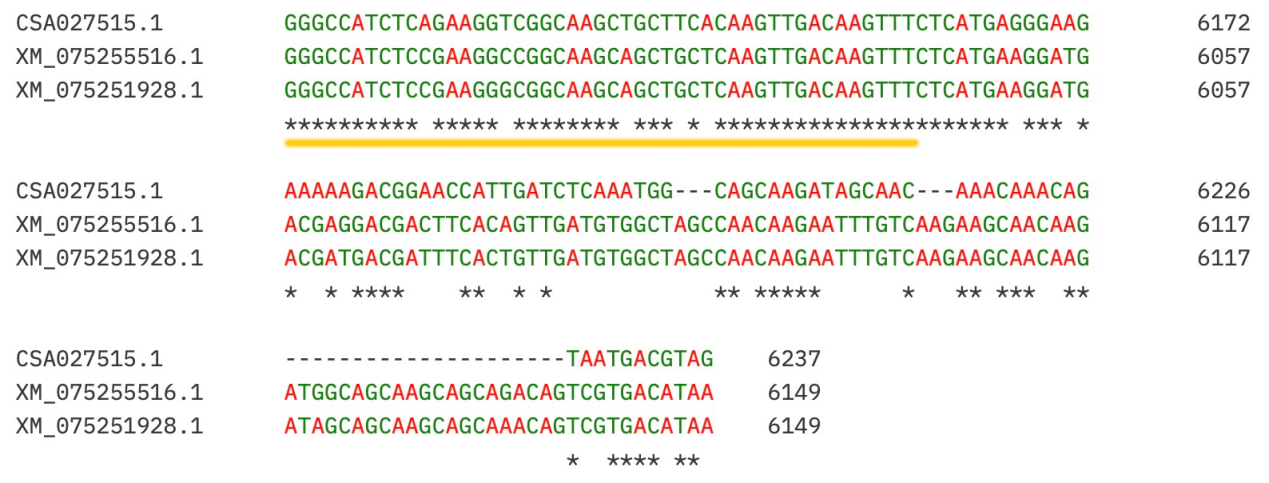


Figure S2 The sequence comparison analysis of *CsNADH-GOGAT* and homologous gene *NtNADH-GOGAT*

Note: CSA027515.1: *CsNADH-GOGAT*; XM_075255516.1 (LOC107809098), XM_075251928.1 (LOC107822887): *NtNADH-GOGAT*; The sequence marked with **blue** underline: miR1507c binding site; The sequence marked with **yellow** underline: NADPH - dependent glutamate synthase beta chain
