# Supplementary figures and images for: LncR12304.1-miR1507c-CsNADH-GOGAT ceRNA module regulates amino acid biosynthesis in tea plant (Camellia sinensis)
Source: Hortic Res. 2026 Jan 13;13(4):uhag014. doi: 10.1093/hr/uhag014 (PMC13095357; doi:10.1093/hr/uhag014)

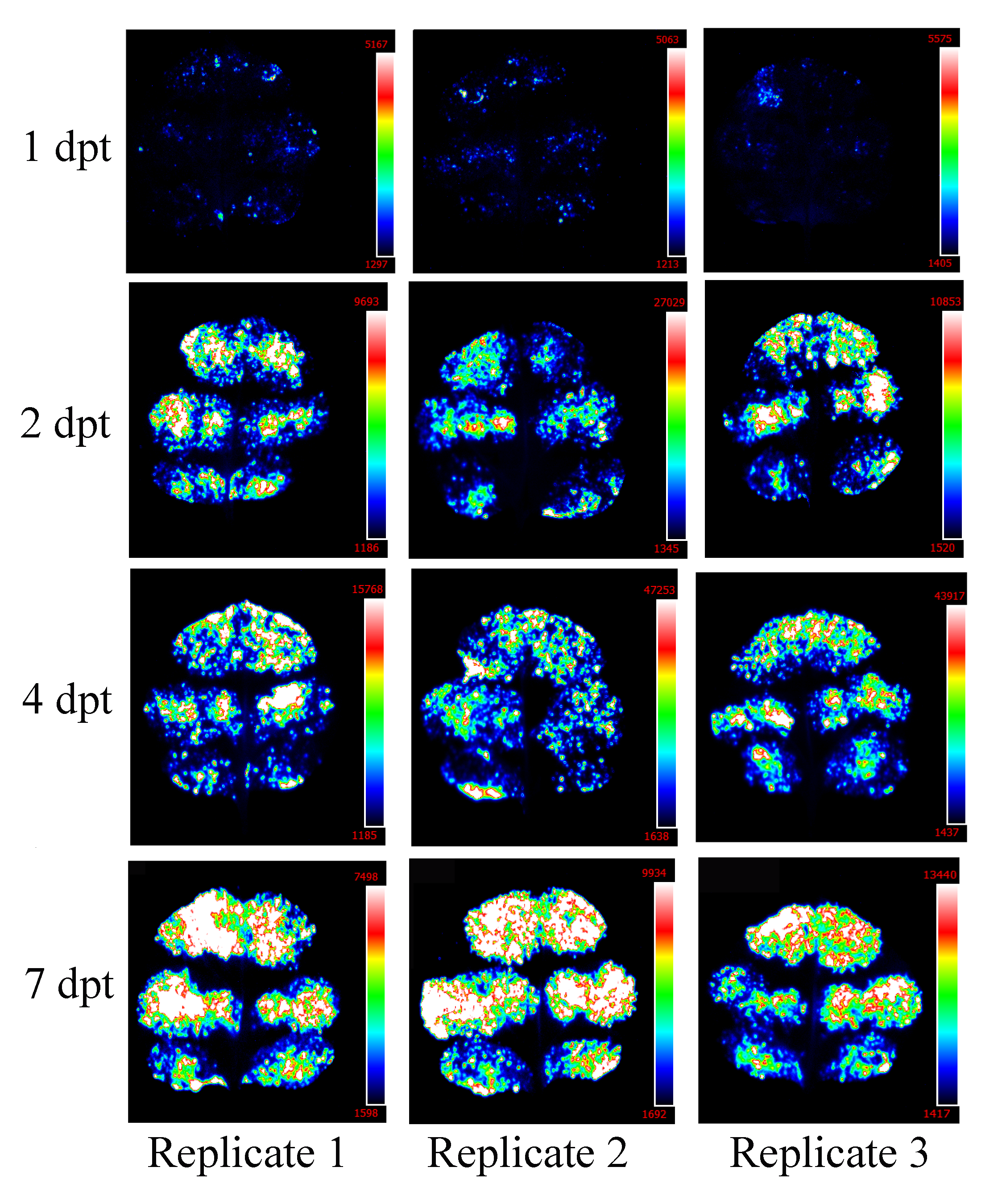

Supplement: Web_Material_uhag014 [file web_material_uhag014.zip › Figure S1.tif]

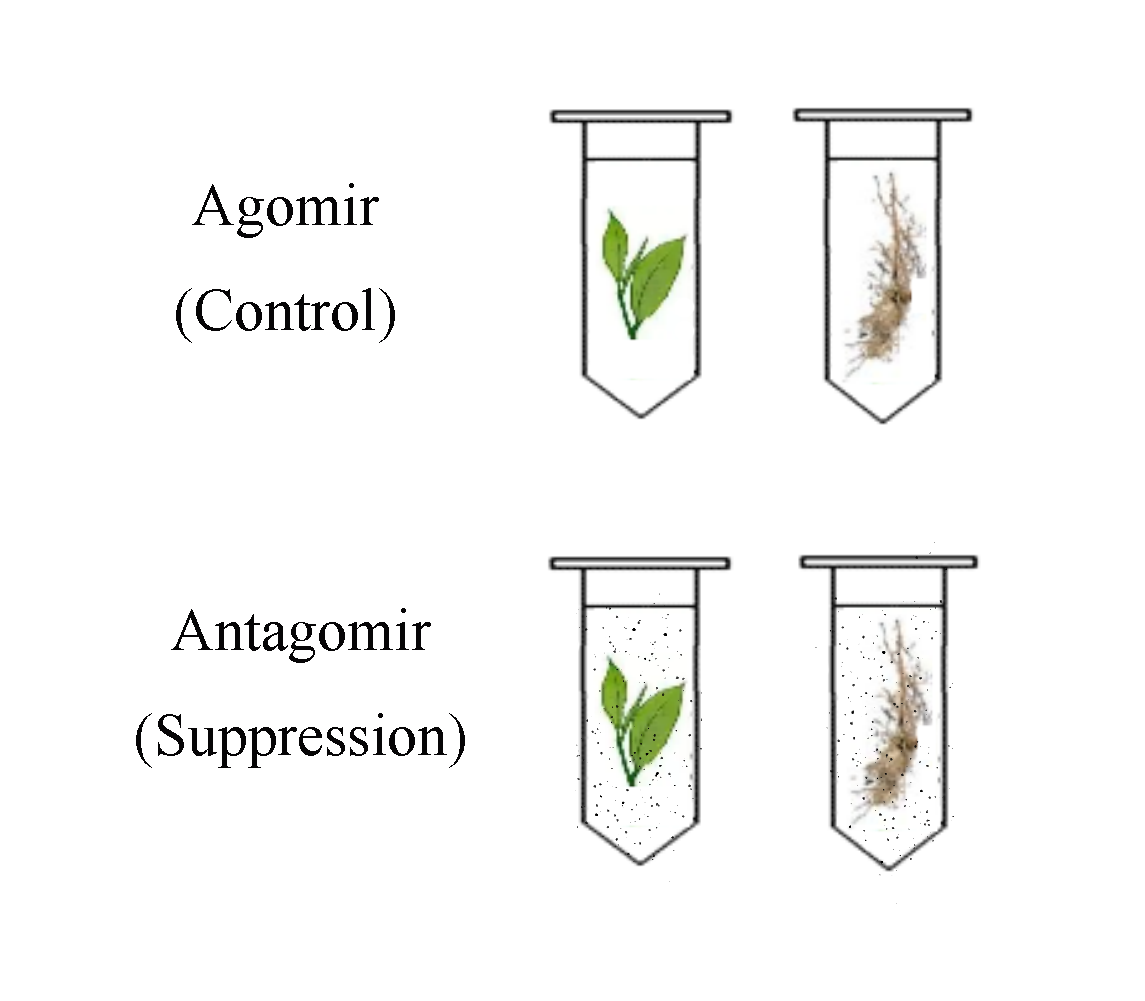

Supplement: Web_Material_uhag014 [file web_material_uhag014.zip › Figure S3.tif]
